# Machine learning for design of degenerate Cas13a crRNAs using lassa virus as a model of highly variable RNA target

**DOI:** 10.1038/s41598-023-33494-4

**Published:** 2023-04-20

**Authors:** T. A. Leski, J. R. Spangler, Z. Wang, Z. Schultzhaus, C. R. Taitt, S. N. Dean, D. A. Stenger

**Affiliations:** 1 Center for Bio/Molecular Science & Engineering, U.S. Naval Research Laboratory, Washington, USA; 2 Present Address: U.S. Department of Agriculture, Riverdale, MD USA; 3 Present Address: Nova Research Inc., Alexandria, VA USA

**Keywords:** Biological techniques, Computational biology and bioinformatics, Microbiology, Molecular biology

## Abstract

The design of minimum CRISPR RNA (crRNA) sets for detection of diverse RNA targets using sequence degeneracy has not been systematically addressed. We tested candidate degenerate Cas13a crRNA sets designed for detection of diverse RNA targets (Lassa virus). A decision tree machine learning (ML) algorithm (RuleFit) was applied to define the top attributes that determine the specificity of degenerate crRNAs to elicit collateral nuclease activity. Although the total number of mismatches (0–4) is important, the specificity depends as well on the spacing of mismatches, and their proximity to the 5’ end of the spacer. We developed a predictive algorithm for design of candidate degenerate crRNA sets, allowing improved discrimination between “included” and “excluded” groups of related target sequences. A single degenerate crRNA set adhering to these rules detected representatives of all Lassa lineages. Our general ML approach may be applied to the design of degenerate crRNA sets for any CRISPR/Cas system.

## Introduction

Rapid and specific detection of infectious agents is necessary for efficient treatment of infectious diseases and epidemiologic surveillance. Nucleic acid-based detection methods such as Polymerase Chain Reaction (PCR), being practically a gold standard for infectious disease diagnostics, offer high specificity and sensitivity but are limited by high cost, the requirement of expensive instrumentation and need for highly trained personnel. Clustered Regularly Interspaced Short Palindromic Repeat (CRISPR)-based detection methods and CRISPR-Associated Protein (CAS) systems emerged recently as an alternative to PCR-based diagnostics with the potential to develop less complex isothermal detection assays but offering the diagnostic accuracy of PCR^1^.

CRISPR detection applications invariably depend on the optimal “design rules” for CRISPR guide RNAs (crRNAs) against specific DNA (DETECTR)^2^ or RNA (SHERLOCK)^3^ targets using Cas12 and Cas13, respectively. Empirical design rules for RNA targets using Cas13 were originally based on observed experimental results of collateral cleavage of fluorescent molecular beacons following specific activation of Cas13 using a relatively small number of candidate crRNAs^4,5^. More recently, Metsky et al.^6^ reported a systematic approach for automated, target-specific crRNA design for large numbers of different pathogens using neural network-based machine learning (ML) algorithms trained on data obtained from massively parallel Cas13a crRNA collateral nuclease screens^7^. That approach depends mostly on selection of multiple specific guide RNAs, usually tolerant of ≤ 2 spacer region mismatches, in order to cover a broad phylogenetic target range.

The concept of degenerate crRNAs for viral detection was reported recently by Barnes et al.^8^ The authors designed and tested a small number of degenerate crRNAs for detection of highly variable Lassa virus (LASV) targets representing lineage II and lineage IV. The crRNA sets were found to be specific for the LASV lineages. That work did not involve testing of comprehensive sets of degenerate crRNAs nor exploration of cross-reactivity with other LASV lineages and with closely related viral species. In another, more recent study by Li et al.^9^ the researchers developed an assay for detection of multiple lineages of Crimean-Congo hemorrhagic fever virus (CCHFV), another highly variable RNA virus, using a single degenerate crRNA. That study demonstrated that the degenerate crRNA used was not cross-reactive with closely related viruses and could tolerate up to 3 mismatches and still activate the collateral RNAse activity of Cas13a enzyme. However, there was no attempt to develop general design rules for application to arbitrary phylogenetic groups.

Several groups have applied ML models to assist in guide design for Cas9-based CRISPR applications that range from simple linear regression models to more complex deep learning models including the bidirectional LSTM (long short-term memory networks) that is part of DeepHF prediction software^10^, or make use of convolutional neural networks^11^. The work presented here was directed at a ML determination of degenerate crRNA spacer sequence attributes that would prove easy to use for prospective design of Cas13a detection assays. This builds on our prior work using high-throughput crRNA screening aimed at finding the common characteristics of crRNA molecules for use in Cas13a based detection assays^12,13^. Our initial goal was to experimentally identify the smallest number of degenerate crRNAs that could activate Cas13a collateral activity to produce a simple binary result for the presence or absence of any member of a phylogenetically diverse group. Subsequently, we applied the RuleFit decision tree machine learning algorithm to find the spacer sequence attributes that determine Cas13a nuclease activity.

Using LASV as a model taxon, we approached this using the following steps:

1. Selection of conserved regions of LASV genomes,
2. Tiled design of degenerate sequences in the complementary spacer regions of crRNAs across those conserved genomic regions,
3. High-throughput screening of the candidate degenerate crRNAs against complementary synthetic targets,
4. High-throughput screening of the selected high performing degenerate crRNAs against targets representing all lineages of LASV (our intended target) and target representing closely related viral species (near-neighbors), and,
5. Use of a machine learning algorithm to analyze the datasets obtained in step (4) to identify the generalizable crRNA design rules for detection of highly variable targets.

Our results demonstrate that besides total spacer region mismatches, the specificity of a degenerate guide set for a selected phylogenetic group depends as well on the spacing of the multiple mismatches relative to each other, the proximity of multiple mismatches to the 5’ end of the spacer and identity of the protospacer flanking sites (PFS). The generality of our ML approach may be applicable to the design of degenerate crRNA sets for other CRISPR/Cas systems.

## Results

### Performance of degenerate crRNA spacer sequences

**Figure 1:**
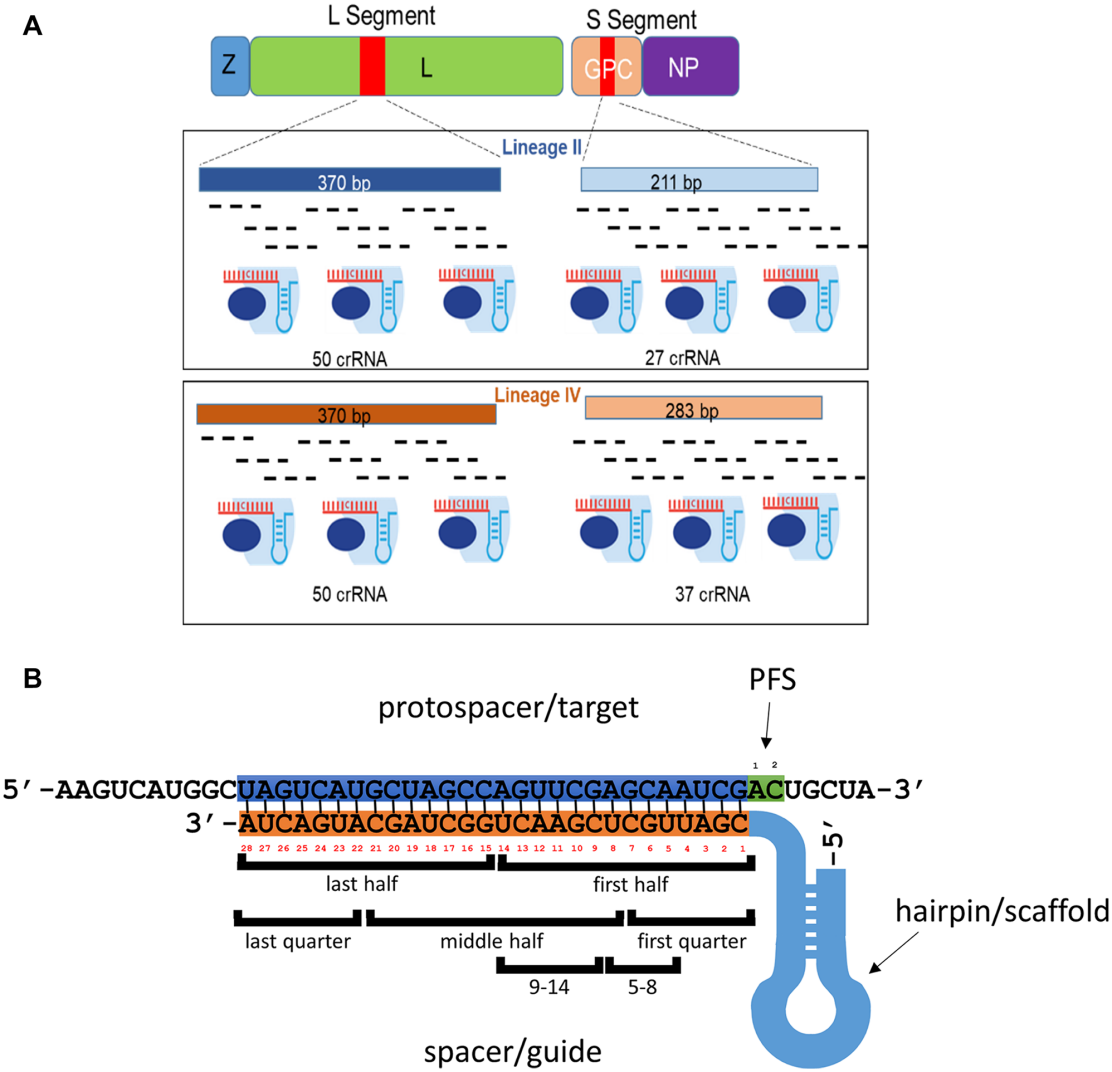
(**A**) Design of the crRNAs targeting conserved regions of LASV L and GPC genes. Overview of the LASV crRNA design strategy. Conserved regions of the L (370 bp) and GPC (211 bp or 283 bp) were selected and consensus sequences for lineage II or lineage IV were constructed (supplementary Table 2 and supplementary materials: lineage II target sequences and lineage IV target sequences). The degenerate spacer sequences of the crRNAs were designed by tiling the 28nt sequences across the consensus target sequences. (**B**) Structure of the Cas13a crRNA and target. Protospacer (target) sequence (top) – blue shading: part of the target complementary to the spacer sequence, green shading: protospacer flanking sites (PFS); crRNA: orange shading: spacer sequence numbered (1–28) in 5′–3′ direction, light blue shading – hairpin/constant part of the crRNA. Location of features defined in RuleFit model are indicated below the spacer sequence.

Our first step was to empirically observe which degenerate crRNAs might allow for high-performance Cas13a-based detection of LASV target sequences located in L and GPC genes. The spacer sequences for large sets of tiled crRNAs contained varying levels of degeneracy. Degenerate sequences are mixtures of all permutations of sequences with alternative nucleotides in degenerate positions. Two types of degenerate nucleotides were used: R (A/G) and Y (C/T). The number describing the degree of degeneracy reflected the number of distinct sequences present in the preparation of a specific degenerate crRNA. The degeneracy of each crRNA used is displayed in Supplementary Figure S3. Four groups of degenerate crRNA sets were tested with their 28 nucleotide (nt) spacer sequences tiled across consensus sequences of conserved regions of each of these genes with separate crRNA sets designed for LASV lineage II and lineage IV sequences (Methods, Fig. 1A).

Performance of the crRNAs was assessed by measuring the total fluorescent signal produced in the Cas13a assay integrated over 2 h in the presence of 3 nM target RNA as described previously^12,13^. The results of the testing are shown in Supplementary Figure S3 and summarized in the Table 1 (averaged background subtracted fluorescence time course data is provided in a separate supplementary file). The crRNAs were grouped into three classes based on the integrated fluorescent signal obtained in relation to the maximum obtained signal for the experiment: high performance (with signal of 80% and higher), intermediate (signal lower than 80% but higher than 20%) and low (signal at 20% or lower). Similar to our earlier studies using crRNAs with non-degenerate spacer sequences designed by tiling along the target sequences^12,13^, the majority of the crRNAs (from 82 to 93% depending on the target sequence) were classified as showing high or medium performance and only a small fraction (from 7 to 18%) of crRNAs produced no or low signal. The number of low performing crRNAs was higher for L targets (12–18%) than for GPC targets (7–11%) and for each of these target sequences the number of low performing crRNAs was higher for lineage IV than lineage II sequences. Importantly, these results indicate that degenerate crRNAs have similar performance to that observed for regular crRNAs (with non-degenerate spacer sequences).

**Table 1:** Results of crRNA performance testing.

Target	Lineage	Number^a^	Performance^b^		
			High	Medium	Low
GPC	II	27	12 (44%)	13 (48%)	2 (7%)
	IV	37	13 (35%)	20 (54%)	4 (11%)
L	II	50	7 (14%)	37 (74%)	6 (12%)
	IV	50	14 (28%)	27 (54%)	9 (18%)

^a^The number of crRNAs tested, ^b^crRNAs were classified into three groups based on the integrated, background subtracted, fluorescent signal relative to the highest signal obtained. Three performance classes were defined: high performance (with signal at 80% or higher), intermediate (signal lower than 80% but higher than 20%) and low (signal at 20% or lower).

### Limit of detection and off-target activity of selected high-performing crRNAs

Each of the degenerate crRNA preparations contained multiple crRNA molecules with variable spacer sequences, where some of the preparations potentially contain over a thousand of different sequences. We conducted multiple experiments in order to find out if such a high level of multiplexing results in detection of non-specific sequences and how it affects the limit of detection (LOD) of the assay.

Four high-performing crRNAs designed for detection of L target (#5_LII, #5_LIV, #9_LII and #9_LIV) were tested for background cross-reactivity and limit of detection (LOD) determination. The degeneracy of these crRNAs ranged from 16 to 1024 (#5_LII – 256, #5_LIV – 512, #9_LII – 16 and #9_LIV – 1024). To test for potential cross-reactivity with non-specific targets, the Cas13a activity assays were performed using high molecular weight human total genomic RNA at 25 ng/µL or high copy short synthetic RNA targets (3 nM *Yersinia pestis lcrV* target RNA) and LASV GPC, lineage IV target RNA at 3 nM. The results of these experiments are shown in Supplementary Figure S4, panels A and B (averaged background subtracted fluorescence time course data is provided in a separate supplementary file). None of these non-specific targets generated any signal above no-target negative controls indicating the lack of off-target activity.

To determine LOD, the Cas13a activity assays for the same set of crRNAs were conducted using tenfold target dilution series ranging from 3 nM to 3 pM. The lowest detectable target concentration was determined to be 30 pM for crRNA #5 and 300 pM for crRNA #9 (Supplementary Figure S5). These results are comparable with previously obtained LODs for non-degenerate crRNAs^13^.

The effect of the genomic RNA background on the limit of detection of the specific LASV L target was evaluated for crRNA #5_LII and #5L_IV (Supplementary Figure S5, averaged background subtracted fluorescence time course data is provided in a separate supplementary file). The results of this assay showed a relatively small decrease in signal (approximately 2 × decrease of fluorescent signal at 30 pM target concentration) in the presence of the genomic RNA background suggesting that background RNA does not interfere to a large degree.

### Detection of LASV lineages and LASV near neighbors

The initial testing of crRNAs was conducted with perfectly matching target RNAs representing LASV lineages II and IV. In order to determine the range of LASV lineages that can be detected by degenerate crRNAs and their potential cross-reactivity with near-neighbor non-LASV sequences, we selected eight high-performing crRNAs designed for detection of L target (#5_LII, #5_LIV, #9_LII, #9_LIV, #29_LII, #29_LIV, #33_LII, #33_LIV). These were tested against a panel of 12 target sequences representing all currently known LASV lineages (I through VII) and eleven near-neighbor Old World arenaviruses (OWA) closely related to LASV (Supplementary Table S1 and Supplementary Figure S1). All target RNAs were synthesized using T7-based transcription of synthetic DNA fragments encompassing the same target region of L gene target described above (Supplementary Table S2 and lineage II and IV sequences in Supplementary Materials).

**Figure 2:**
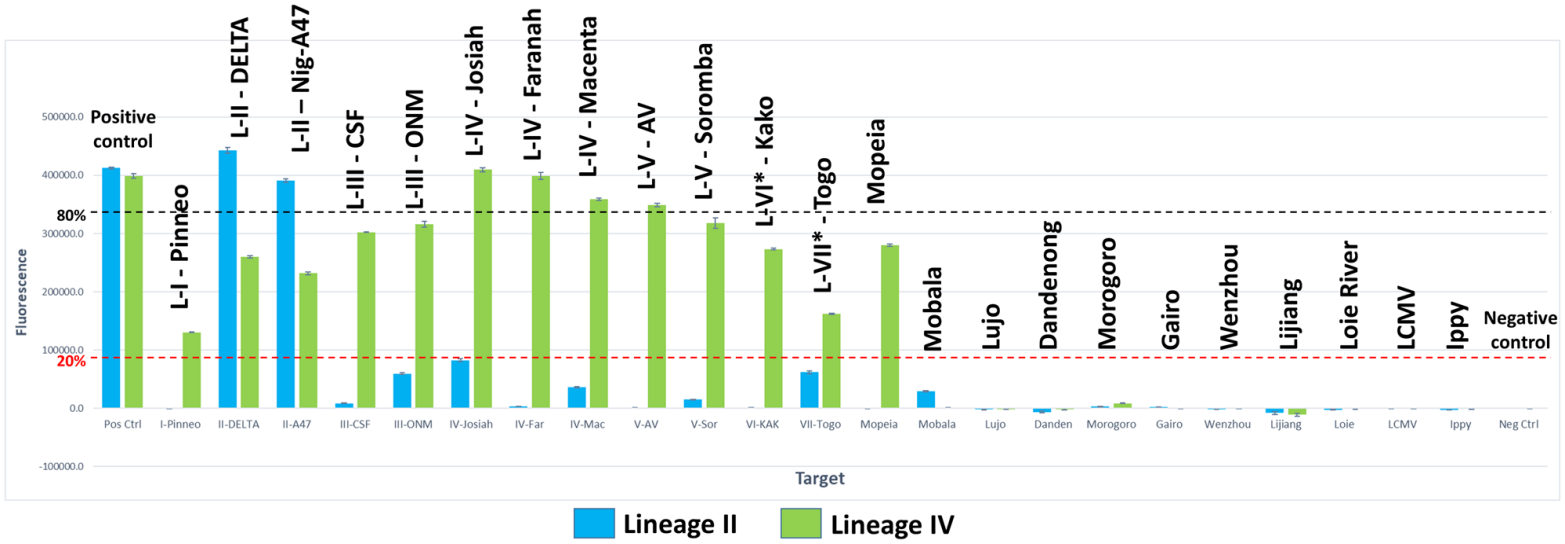
crRNA #5—detection of LASV lineages and near-neighbors. Results of testing of crRNAs #5_LII and #5_LIV with a panel of 12 LASV targets representing all currently known lineages (I–VII) and 11 near-neighbor old world arenavirus (OWA) targets. The height of the bars reflect the cumulative background subtracted fluorescence obtained for each of the tested crRNAs against the specific target.

Testing of the eight degenerate crRNAs was performed using the same Cas13a activity assay as described above for all panels of crRNA molecules. The results are summarized in Table 2. Detailed results for crRNA #5_LII and crRNA #5_LIV are shown in Fig. 2 and results for all eight crRNAs in Supplementary Figures S6 through S9 (averaged background subtracted fluorescence time course data is provided in a separate supplementary file). The assay was considered positive for detection of a particular target if the generated integrated fluorescent signal was > 20% of the maximum observed signal which includes medium- and high-performing crRNAs. crRNA #5_LIV was found to be the best crRNA for discrimination between LASV and its near-neighbors with all 12 LASV targets and only one out of 11 near-neighbors classified as positive. In general, the crRNAs #5_LII, #5_LIV, #9_LII and #9_LIV showed relatively good discrimination between LASV and near neighbor targets. For these crRNAs, at most one near neighbor was classified as positive while detection of LASV was more varied with 2 to 12 out of a total of 12 targets classified positive. For the crRNAs #29_LII, #29_LIV, #33_LII and #33_LIV, higher numbers of near-neighbor targets (from 3 to 5 out of 11) were positive with the exception of crRNA #33_LIV, in which case no near neighbors gave a positive result. For crRNAs #29_LII, #29_LIV, #33_LII and #33_LIV, several of LASV targets (between 1 and 9) were not detected.

**Table 2:** Detection of L gene of LASV lineages and near-neighbors.

crRNA	Lineage	LASV lineages (n = 12)		Near-neighbors (n = 11)	
		Positive^a^	Negative^b^	Positive^a^	Negative^b^
#5	II	2	10	0	11
	IV	12	0	1	10
#9	II	9	3	1	10
	IV	11	1	1	10
#29	II	9	3	4	7
	IV	11	1	5	6
#33	II	3	9	3	8
	IV	4	8	0	11

^a^The assay was classified as positive if the integrated, background subtracted, fluorescent signal was higher than 20% of the highest signal obtained.

^b^The assay was classified as negative if the integrated, background subtracted, fluorescent signal was equal or lower than 20% of the highest signal obtained.

These results indicate that the range of detected targets was related to the specific sequences of the particular crRNAs and in some cases allowed for detection of all LASV lineages tested.

### Impact of spacer/target pairing properties on Cas13a activity

**Figure 3:**
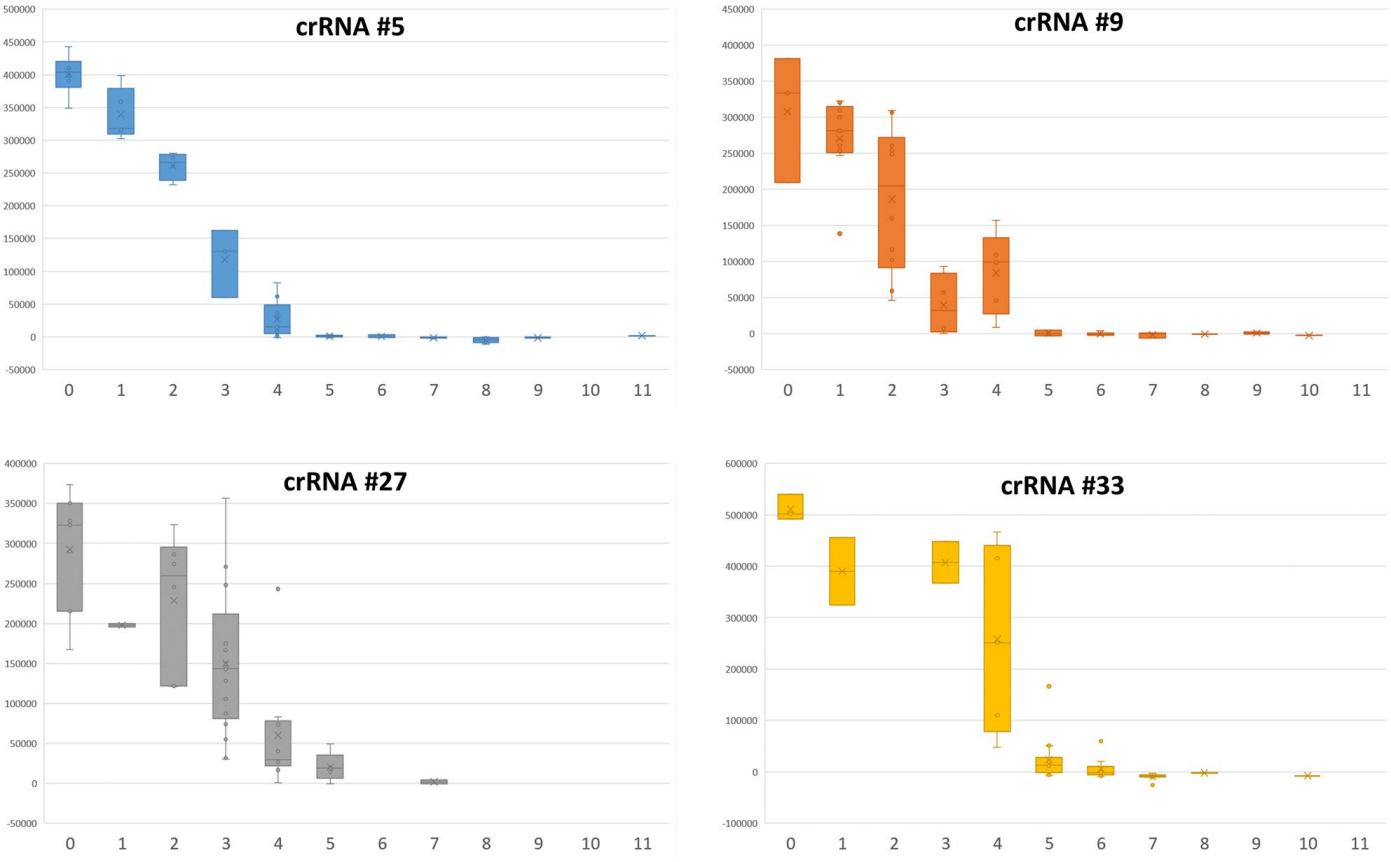
Relationship between spacer/target mismatch number (x-axis) and Cas13a assay fluorescent signal (y-axis). Relationship between the number of mismatches of the spacer/target pairs (for Watson–Crick pairing rules) and the cumulative background subtracted fluorescence signal obtained in Cas13a assay for all the spacer/target pairs tested for L gene crRNAs #5, #9, #29 and #33.

All eight tested degenerate crRNA spacer sequences were aligned with all target sequences to determine the impact of the number and positions of mismatches on the outcome of the Cas13a activity assays. The mismatches were identified using three different sets of criteria (Watson–Crick, symmetric G-U wobble and asymmetric G-U wobble). Figure 3 plots the number of mismatches against the fluorescent signal for all the crRNAs tested (results for crRNAs testing for both lineages were plotted together). The plots in Fig. 3 were created using the Watson–Crick mismatch dataset (for plots based on G-U wobble datasets see, Supplementary Figure S10). The plots in Fig. 3 show that the total number of mismatches inversely correlates with the average strength of the fluorescent signal. For Watson–Crick pairing, the majority of the spacer/target pairs with three or less mismatches produced sufficient fluorescent signal to classify them as positive (for G-U wobble mismatch datasets this number decreased to two mismatches, shown in Supplementary Table S5). For spacer/target pairs with five or more mismatches, the fluorescent signal was always classified as negative (with one exception) and spacer/target pairs with exactly four mismatches contained a mix of pairs classified as positive and negative (for Watson–Crick dataset).

**Figure 4:**
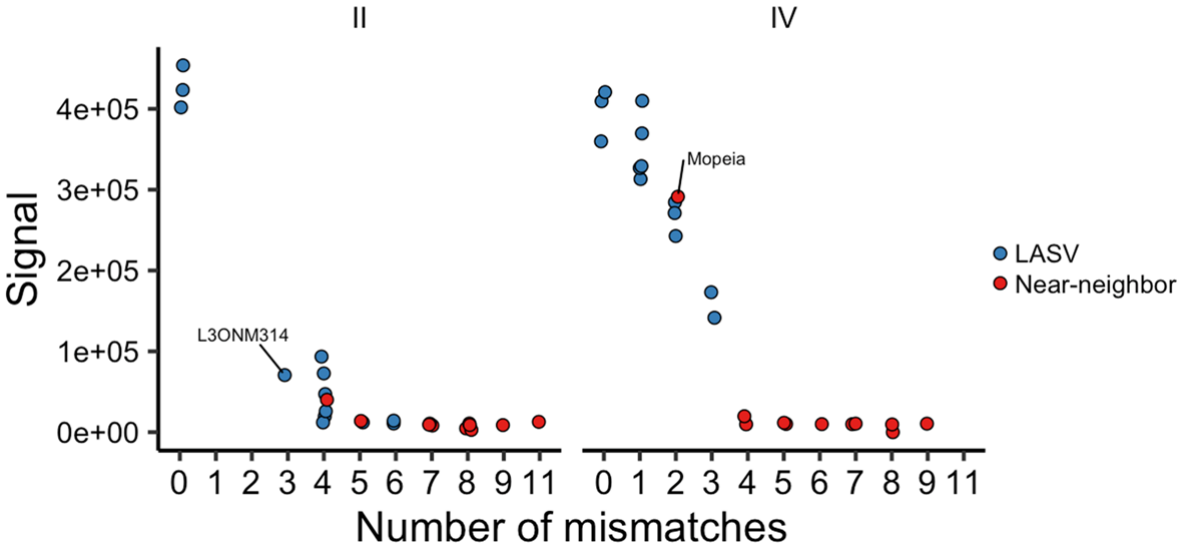
Fluorescent signal obtained for crRNA #5 spacer/target pairs with different numbers of mismatches. The circles represent all spacer/target pairs tested for crRNA #5_LII (left panel) and #5_LIV (right panel). While generally higher number of mismatches correlates with lower signal, the pairs with the same number of mismatches (e.g. 3 mismatches) can have variable signal depending on other features of the pairing (mismatch distribution—IQR, PFS nucleotides, etc.).

Figure 4 shows a comparison of signal intensities for crRNA #5 guides against all targets. Considerable variability in signal was observed for two, three and four total mismatches, indicating that variables other than total number of mismatches have important effects on the signal intensity. To understand how the distribution of mismatches and other variables influence the outcome of the detection assays, a RuleFit algorithm-based predictive model was developed using our experimental data. The RuleFit model described here fits into the low to medium complexity range of the Type III Learning-based category, as defined by Konstantakos et al. for Cas9 CRISPR applications, where CRISPRscan uses a linear regression model at the low end and several deep learning models have been applied to guide design on the high end^11^.

**Figure 5:**
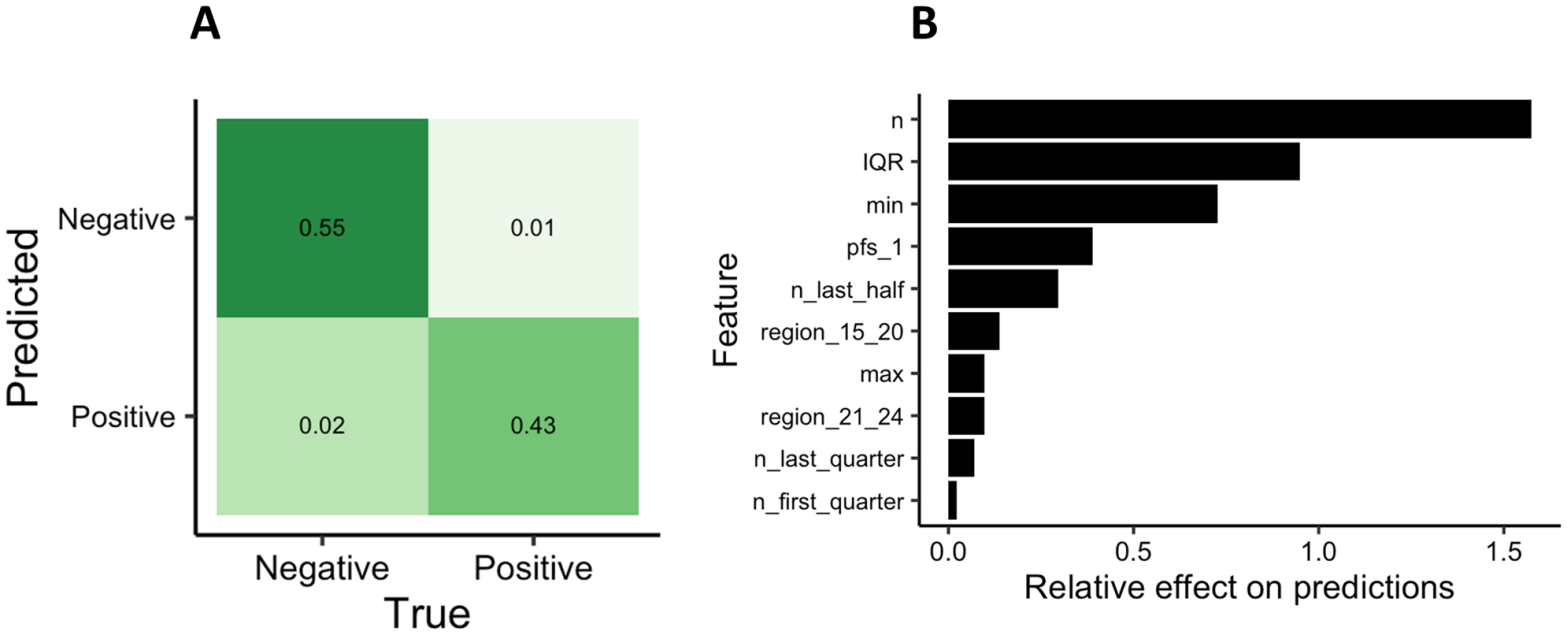
Model performance and the most important features. RuleFit classifier model performance for Watson–Crick base pairing. (**A**) Confusion matrix showing percentages of actual assay outcomes versus outcomes produced by the classifier model, (**B**) relative effect of features on model predictions: n—number of mismatches, IQR—interquartile range for mismatch positions (characterizes distribution of mismatches with IRQ = 14 indicating even distribution and IRQ > 14 and IRQ < 14 indicating clustering), min—position of mismatch closest to 5’ end of crRNA, PFS_1—nucleotide at protospacer flanking site #1, range—distance between the furthest apart mismatches, remaining features corresponding to various regions of crRNA as described in Fig. 1B.

The RuleFit classifier was trained to predict whether a guide/target pair would yield a signal above or below threshold (defined as 20% of maximum signal) using the three mismatch datasets (based on Watson–Crick pairing or two versions of G-U wobble pairing). The output accuracy after tenfold cross-validation was 95.0% ± 2.0% for the Watson–Crick pairing dataset. The model had similar accuracy using the G-U wobble datasets (95.4% ± 1.4% for asymmetric G-U wobble and 93.6% ± 1.9% for symmetric G-U wobble). A representative confusion matrix of the predictions for Watson–Crick pairing dataset is shown in Fig. 5. The confusion matrix (Fig. 5, panel A) compares the predictions of the model with the actual experimental data. The model shows more false negatives than false positives with an overall accuracy of 95% (for the Watson–Crick dataset, see Supplementary Figures S12, panel A and S14 panel A for confusion matrices for G-U wobble datasets). To look at the broader range of model threshold values, we also generated Receiver Operating Characteristic (ROC) curves for all three datasets and calculated Area Under the Curve (AUC) values. The AUC for Watson–Crick pairing dataset was 0.97, for asymmetric G-U wobble 1.0 and for symmetric G-U wobble 0.97 (Supplementary Figures S10, S13 and S15).

Using this model, we interrogated the rules for feature importance (see Fig. 5, panel B for Watson–Crick dataset and panel B of Supplementary Figures S12 and S13 for G-U wobble results). As expected, the feature with the overall highest global impact on the predictions for all three datasets was the total mismatch count (n). The analysis of the signals obtained for crRNA #5 (Fig. 4) shows that for spacer/target pairings with the same number of mismatches the signal can be significantly different indicating importance of other features for some mismatch ranges. The second most significant feature for all datasets was the interquartile range (IQR,) which reflects the distribution of mismatches across the spacer. IQR indicates whether the mismatches are clustered or are evenly distributed along the spacer sequence. For the Watson–Crick pairing, the position of the 5’-most mismatch (min) and the identity of the nucleotide located at the protospacer flanking site #1 (PFS_1) also influenced the prediction. For the symmetric G-U wobble pairing dataset, the protospacer flanking site #1 (PFS_1) had the third highest influence on prediction following total mismatch number and IQR, each exceeding 0.1, while the mean value calculated for the spacer position of all mismatches (mean) was the only other feature with a value exceeding 0.1 for the asymmetric G-U pairing dataset. The most important regions for mismatch count were the last quarter of the guide (or positions 21–28), first half of the guide (positions 1–14), and the region 5–8. These last two regions overlap with the mismatch hypersensitive “center seed” region (bases 8 to 16) found previously to be essential for initial target binding and the HEPN-nuclease switch 5–8 region responsible for inducing collateral RNase activity^5,14^. While the exploration of mechanism of target RNA binding and Cas13a RNase activation was outside of the scope of this study, the obtained results were generally in line with the previous findings. For example, no spacer/target pair with three consecutive mismatches in the “seed region” was found among pairs classified as positive based on Cas13a activity assay. Overall, the properties of the analyzed features suggest that, while overall mismatch count (n) was the most important, the location and distribution of those mismatches and identity of a nucleotide at PFS #1 had the strongest relative impact on classification (i.e., assay outcome).

The RuleFit classifier model identifies the prediction rules associating the most important features. For some crRNAs like #5, the classification is almost perfectly correlated with number of mismatches (n). For crRNA #5 spacer/target pairings, 17 out of 18 with n < 4 are positive while for pairings with n ≥ 4, all 30 out of 30 are negative. However, more discriminating rules involving more features are needed for predicting classification of other spacer/target pairing. For example for all spacer/target pairs with 3 mismatches, only 73% have positive signals. After filtering this group using IQR ≥ 6.25, 89% are positive. Finally, after selecting only the guides where PFS_1 is not G, a 100% positive rate is achieved.

The top ten rules identified for the Watson–Crick dataset are provided in Supplementary Table S4. For example, the listed rules containing IQR suggest that a more even distribution of mismatches was important for classification as a positive signal and, conversely, several mismatches within a small area will likely lead to classification as a negative signal.

**Figure 6:**
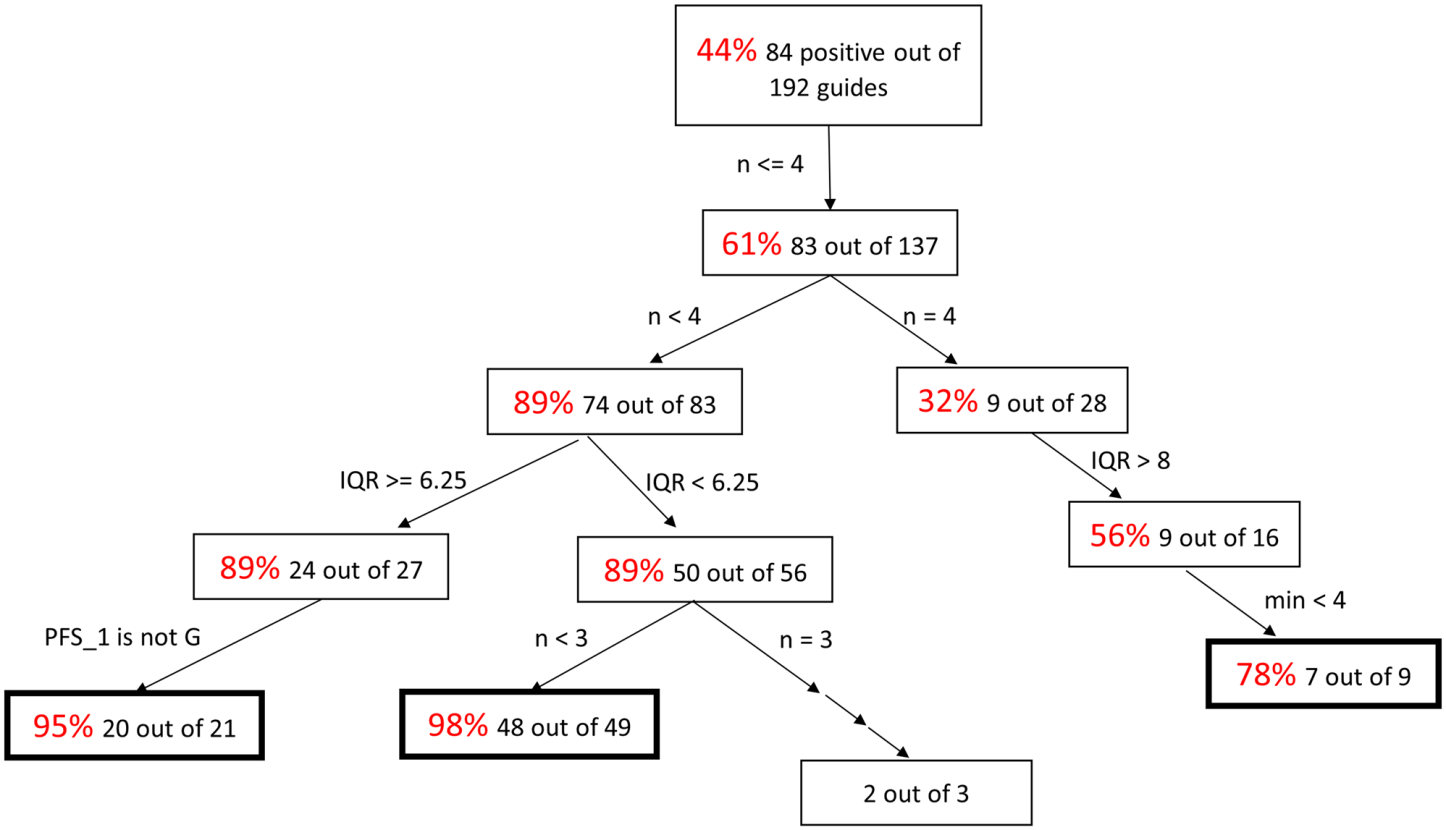
Maximizing guides with positive signals and recommended feature ranges (Watson–Crick). Decision tree for maximizing the number of guides with positive signals. Percentage of positive guides for each set of conditions is highlighted in red. Using the rules output by the RuleFit classifier, conditional control statements were used to filter the initial 84 positive guides from the total set of 192. The features used are n, IQR, min, and PFS_1. Linearized, the decision rules for maximizing positive signals in our set of guides are 1) if IQR is ≥ 6.25, n is between 0 to 3 and pfs_1 is A, U, or C, 2) if IQR is < 6.25, n is between, 0 to 2, and 3) if n = 4, IQR is between 9 and 28 and min is between 1 and 3. These rules achieve 95% positive guides from an initial pool that is 44% positive, including guides with up to four mismatches.

An example decision tree used to generate prediction rules is shown in Fig. 6. The features used are n, IQR, min, and PFS_1. Linearized, the decision rules for maximizing positive signals in our set of guides are: 1) if IQR is ≥ 6.25, n is between 0 to 3 and pfs_1 is A, U, or C, 2) if IQR is < 6.25, n is between, 0 to 2, and 3) if n = 4, IQR is between 9 and 28 and min is between 1 and 3. These rules achieve 95% positive guides from an initial pool that is 44% positive, including guides with up to four mismatches.

## Discussion/conclusions

Nucleic acid amplification based detection technologies (e.g., PCR, LAMP and many others) rely on recognition of target sequences by specific primers. In the case of highly variable targets, finding the primers sets without mismatches to at least some variants of the target may be challenging^15^. One way to solve this issue may include multiplexing or use of degenerate primers^16,17^. The goal of this study was to assess the utility of crRNAs with degenerate spacer sequences for detection of highly variable RNA targets using the Cas13a based CRISPR detection assay. We used LASV sequences as a model of a highly diverse taxon. LASV, a viral pathogen and an etiologic agent of a highly lethal hemorrhagic fever (Lassa fever—LF)^18^, has always posed difficulty for the development of nucleic acid based assays due to its high variability with close to 30% divergence on the RNA level^19,20^. An alternative approach using “universal” crRNA for Cas9-based editing of polymorphic genes was shown by Krysler et al.^21^ That approach utilized nucleotides such as inosine (which pairs with all four RNA nucleotides). In comparison, our approach uses degenerate crRNA sets comprised of standard RNA nucleotides. Our work is confirmatory of other Cas13a crRNA design assumptions that 2 and 3 spacer mismatches are tolerated for specific (Metsky et al.^6^) and degenerate (Barnes et al.^8^, Li et al.^9^) crRNAs, respectively. We show for the first time that in order to rigorously extend the use of degenerate crRNAs for inclusion of diverse phylogenies, other determinants of spacer sequence effectiveness and specificity must be considered.

The immediate utility of degenerate crRNAs lies in the minimization of guides needed whenever fine discrimination of a diverse taxon is not required and the number of crRNAs (with corresponding sensor bandwidth) must be minimized, most notably in handheld or lateral flow assay formats. Our results show that this can be accomplished without loss of sensitivity or cross-reactivity with high levels of background RNA. The specific feature ranges will likely be dependent on tradeoffs between specificity and signal levels. In this study, we arbitrarily defined threshold signal as > 20% of maximum at 2 h. In practical terms, the design feature ranges should be comparable when using initial signal rates versus final signal levels and different threshold signal percentages.

A generalized approach for predictive application of our design rules to Cas13a activation (Supplementary Figure S16):

1. Assemble and align representative set of sequences for both “inclusion” (taxon on interest) and “exclusion” (near neighbors for which sub-threshold signals are required);
2. Scan genome assemblies of “inclusion” sets to locate candidate nucleotide regions that can be populated with degenerate nucleotides to cover the entire inclusion set while still satisfying degenerate guide RNA design rules. If multiple guides are needed for a given inclusion taxon, identify those.
3. Apply guide design rules to identify any possible false positive signal generation by scanning “exclusion” taxon sequences. Eliminate those candidate guides if detected.
4. Perform limited high-throughput screening of remaining candidate degenerate guides to experimentally confirm signal generation and lack of cross-reactivity.

The RuleFit based classifier presented in this study was validated using sequences of only one highly variable target represented by LASV and its close neighbors. To ascertain that the algorithm is not biased towards specific patterns present in LASV sequences and is more generally applicable, testing with other highly variable targets will be needed. Another limitation of the study is that only guides having relatively fixed composition (constant spacer and hairpin lengths) were screened for their ability to activate LwaCas13a collateral nuclease activity at greater than an arbitrarily-defined threshold, as measured following the completed reactions with molecular beacons. Specific applications may require shorter reaction times as well as different arbitrary cut-off thresholds for collateral activation. These parameters can be explored using the complete reaction data set (available at https://github.com/NRL-CRISPR/CRISPR-rules). Application of ML to different CRISPR/Cas protein combinations can in principle be readily achieved using analogous experimental and ML methods described herein.

## Methods

All experimental procedures described in this manuscript were carried out in accordance with relevant guidelines and regulations including biosafety and chemical safety regulations. All nucleic acid sequences used were obtained from publicly available NCBI/GenBank collections and no human subject research was conducted in the course of this project. No materials classified as select agents were obtained or used in any experiments described in this manuscript.

### Design of LASV target sequences and degenerate crRNA sequences and PCR primers

Due to a very high overall diversity of naturally occurring LASV sequences (see Supplementary Materials: LASV virus lineages), the initial target sequences used in this study were limited to sequences from the two most widely circulating LASV lineages: lineage II, the dominant lineage circulating in Nigeria and lineage IV, the lineage circulating in Mano River Union region (MRU, Guinea, Liberia and Sierra Leone). Other lineages (I, III and V through VII) are relatively rarely isolated and currently represented by a small number of sequences in GenBank. Regions of GPC and L genes with relatively conserved sequences were selected as the targets (Supplementary Table S2). The same regions were used in the past in the RT-PCR based assays for LASV detection^22^. We decided to target these regions to minimize variability of the targets and maximize the likelihood of designing guide sequences with minimal level of degeneracy.

### Degenerate target sequences and design of degenerate crRNAs

The degenerate consensus sequences for crRNA spacer design were generated as follows. For the lineage II, four partial GPC and L sequences from human isolates from Nigeria were aligned. For the lineage IV, 35 partial GPC and L sequences obtained from both human and rodent LASV isolates from MRU were aligned (Supplementary Table S2). The alignments for both lineages were performed with ClustalW algorithm using MEGA software^23^. The degenerate nucleotides (A/G: R (purine), C/T: Y (pyrimidine)) were manually identified in the aligned sequences. While other types of degenerate nucleotides are available (e.g., W (weak): A/T, S (strong): C/G and several others) there was no need to use other kinds of degenerate bases for the consensus sequences obtained in this study. Consensus target sequences for the crRNA spacer sequence design were created by using the isolate Nig08-A37 sequences for lineage II targets and isolate SL15 sequences for lineage IV targets as the base sequences. The polymorphic positions identified in the alignments were replaced with degenerate bases either R (A/G) or Y (C/T)—(Supplementary Table S2 and Supplementary Materials: lineage II target sequences and lineage IV target sequences). Then the 28 nt spacer oligonucleotides were defined as 21 nt overlapping sequence fragments and tiling across the entire target regions (Fig. 1A). Sequences of the DNA oligonucleotides encoding crRNAs were designed by adding the variable spacer sequences to the 5’ end of the backbone sequence (direct repeat sequence) and T7 polymerase promotor sequence to the 3’ end of the backbone as reported previously (Supplementary Materials: Sequences)^13^. The degeneracy of crRNAs used in this study ranged from 2 (1 degenerate base) to 4096 (12 degenerate bases), Supplementary Figure S3.

### Synthetic DNA for target RNA production

The synthetic DNAs used to produce the RNA target sequences for use in the Cas13a activity assays were extended versions of the target regions described above and were based on the same Genbank base sequences (Supplementary Table S2 and Supplementary Materials: lineage II target sequences and lineage IV target sequences). The PCR primers complementary to the flanking sequences of the target regions were designed for each of these sequences. T7 RNA polymerase promoter sequences were added to 5’ end of the forward primer in each primer pair (Supplementary Materials: Sequences).

### Other LASV and near neighbor targets

Sequences of L gene target region of twelve LASV isolates representing all currently known lineages and eleven arenavirus species closely related to LASV (near neighbors) were selected for testing the selected highly performing crRNAs. Synthetic DNA fragments of the L gene covering the same region as the DNA fragment were used for synthesis of lineage II L target RNA described earlier. PCR primers with T7 RNA polymerase promotor built-in were also designed for each of these sequences as described above. See Supplementary Table S1 for the summary information and Supplementary Materials: Sequences for DNA fragment and primer sequences.

### Synthesis of degenerate crRNA and RNA target sequences

The degenerate crRNA molecules were obtained by conducting in vitro transcription of crRNA encoding DNA oligonucleotides described above. The oligonucleotides were purchased from Eurofins Genomics (Louisville, KY), and listed in Supplementary Materials: Sequences. In vitro transcription was done using the HiScribe™ T7 Quick High Yield RNA Synthesis Kit (New England Biolabs, Ipswich, MA). The transcription reactions were conducted in high-throughput format using the strategy described earlier^12^. The individual transcription reactions were performed in 25 μL total volume. This included 0.5 μL of 100 μM T7 forward primer, 1.5 μL of 100 μM crRNA-encoding DNA oligonucleotide, 1.25 μL of T7 RNA polymerase, 9.25 μL of 2 × NTP buffer and 12.5 μL of nuclease-free ddH_2_O. The reactions were carried out for 2 h at 37 °C. The obtained crRNAs were used in Cas13a activity assays without additional purification. This approach has been validated in two prior studies^12,13^.

Target RNA sequences were also prepared using HiScribe™ in vitro transcription system. Fragments of LASV GPC or L genes were amplified, using primers listed in Supplementary Materials: Sequences, using FastStart Taq DNA polymerase kit (Millipore-Sigma, Burlington, MA, USA) according to the manufacturer’s instructions. The forward PCR primers included T7 promotor sequences, which were incorporated into the amplicons.

The transcription reactions were set up using 2 μL of unpurified DNA amplicon preparation, 2 μL of T7 RNA polymerase, 10 μL of 2 × NTP buffer and 16 μL of nuclease-free ddH_2_O (30 μL of total reaction volume). The transcription reactions were incubated at 37 °C for 2 h after which 5 μL Turbo DNAse (ThermoFisher, Grand Island, NY) and 15 μL of nuclease-free ddH_2_O were added (increasing the total volume to 40 μL) and incubated further 30 min at 37 °C to remove the template DNA. The obtained transcript preparations were cleaned up using using RNA Clean and Concentrator 25 kit (Zymo Research, Irvine, CA USA) according to the manufacturer instructions. The RNA concentration was determined using Qubit fluorometer and RNA BR (broad range) assay kit (ThermoFisher). The template solutions were diluted to 150 mM for use in Cas13a activity assays.

### High throughput crRNA performance testing using Echo acoustic liquid handler

Both crRNA synthesis and Cas13a activity assays were conducted using a high throughput workflow in 384 well plates with fluid transfer handled by Echo 525 acoustic liquid handler (Beckman Coulter, Indianapolis, IN) using the Plate Reformat software provided by the manufacturer as described earlier^12^, Supplementary Figure S2.

In order to generate crRNAs, 27–50 crRNA transcription reactions were set up using reagent volumes as described above for an individual reaction. The reactions included the tested crRNAs and a negative control (crRNA template oligo replaced with TE buffer). First, master mix containing all reaction components except for template oligonucleotide were distributed using Echo instrument from 6 well Echo qualified reservoir plate (cat# ER-0050) to Echo qualified 384 well microplate (cat# ER-0050). 23.5 μL of the master-mix were transferred to each well. Subsequently 1.5 μL of the crRNA template oligonucleotides were added to each well containing master-mix using Echo instrument from a previously prepared Echo qualified 384 well microplate. The plates were spun briefly in a centrifuge at approximately 1500 *g* to bring all the liquid to the bottom of the wells and remove air bubbles. The plate was sealed using MicroAmp Clear Adhesive Film sealer (ThermoFisher) and incubated for 2 h at 37 °C. After incubation, the plates with transcribed crRNAs were stored in − 80 °C. For long term storage MicroAmp sealers were replaced with Adhesive PCR Sealing Foil (cat# AB-0626, ThermoFisher).

To determine the efficacy of each crRNA, Cas13a nuclease activity assays were conducted using Cas13a enzyme from *L. wadei*^4^ which was synthesized and purified by GenScript Biotech (Piscataway, NJ). The enzyme was stored and diluted using the storage buffer (50 mM Tris–HCl, 600 mM NaCl, 5% Glycerol, 2 mM DTT, pH 7.5). Each nuclease activity assay was performed in 20 μL reaction that included 1 μL of 1 μM Cas13a, 1 μL of 2 μM RNase alert v.2 (from RNaseAlert™ QC System v2, ThermoFisher), 17.2 μL of nuclease assay buffer (40 mM Tris–HCl, 60 mM NaCl, 6 m M MgCl_2_, pH 7.3), 0.4 μL of crRNA (from unpurified transcription reaction) and 0.4 μL of 150 mM target RNA. For each crRNA a total of six reactions were set up, with three target negative reactions and three target positive. First, master mix containing all reaction components except for crRNA and target RNA were distributed using Echo instrument from 6 well Echo qualified Reservoir plate to a 384 well assay plate (black with clear flat bottom, cat#3762, Corning Life Sciences, Tewksbury, MA). A total volume of 19.2 μL of the master-mix was transferred to each well. Next, 0.4 μL crRNAs from previously prepared 384 well microplate were transferred using Echo instrument to the wells containing the master-mix in such a way that each crRNA was added to 6 subsequent wells in the reaction plate. Finally, 0.4 μL of the target RNAs (previously placed in the area of the crRNA plate not occupied by transcribed crRNAs) were added to three of the wells for each crRNA. The Cas13a reaction plates were spun briefly in a centrifuge at approximately 1500 × *g* to bring all the liquid to the bottom of the wells and remove air bubbles. Immediately after spinning, the reaction plates were sealed using the MicroAmp sealers. The plates were incubated in Biotek Synergy Neo2 plate reader (Biotek, Winooski, VT) at 37 °C and fluorescence was read from the bottom of the wells every 5 min for 2 h using excitation at 490 nm, emission at 520 nm and gain set at 100.

The integrated background corrected final fluorescence values reflecting the Cas13a RNase activation for each of the crRNAs was calculated by subtracting the sum of averages of fluorescence measured for template negative samples over the course of the experiment (25 measurements) from sum of averages for template positive samples.

The crRNAs were classified into three groups based on the integrated, background subtracted, fluorescent signal relative to the highest signal obtained. Thee performance classes were defined: high performance (with signal at 80% or higher), intermediate (signal lower than 80% but higher than 20%) and low (signal at 20% or lower).

### Dataset, data processing, and feature extraction

A dataset was constructed based on the results of a series of assays testing the performance of eight selected crRNAs (#5, #9, #29 and #33, lineage II and IV versions for each of these crRNAs) designed for detection of LASV L target with a panel of targets containing 12 LASV sequences representing all known LASV lineages and 11 viral species closely related with LASV. Each data entry included a list of positions of mismatches between the crRNA spacer and the corresponding target sequence together with a fluorescent signal obtained in the Cas13a activity assay using this crRNA spacer/target combination. To identify the mismatch positions the target sequences (converted to DNA sequence) and reverse complements of the crRNA spacers containing degenerate residues (also converted to DNA sequences) were compared. Mismatches were identified by first decomposing R and Y degenerate bases to A/G and C/T, respectively, based on IUPAC definitions^24^, and then applying binary labels for match/mismatch for each base and each spacer/target pairing.

For the baseline mismatch dataset the mismatch positions were determined according to the standard Watson–Crick pairing rules. However, while we used the DNA versions of the spacer and target sequences for the mismatch determination the actual molecules interacting in the Cas13a activity assays are RNA molecules. It was found that in RNA-RNA pairing the so called “G-U wobble” pairing has a similar thermodynamic stability to the Watson–Crick base pairs (G-C and A-U in case of RNA)^25^. However, the results of a recent study on spacer/target interaction in CRISPR-Cas13a system the G-U pairs functioned as a match only in the configuration in which G was in the spacer and U in the target sequence^6^. For the above reasons we created two additional mismatch datasets: one treating all the pairings corresponding to G-U pairs in RNA sequences as a match (symmetric G-U wobble dataset) and another in which the pairings corresponding to the G-U wobble were treated as a match only in the cases when G was in the crRNA spacer and U was in the target (asymmetric G-U wobble dataset). Each of the final datasets had 192 entries.

For each of the dataset entries 22 features were extracted or calculated from the mismatch data and target sequences. The features describe various aspects and properties of the specific spacer/target pair. The detailed description of the features is summarized in the Supplementary Table S3 and the locations of certain of these features in the crRNA are shown in Fig. 1B. The features include the total number of mismatches (n) and several features characterizing the location and distribution of these mismatches. They include features specifying number and frequency of mismatches in certain regions of the spacer (n_first_half, n_middle_half, n_last_half, n_first_quarter, n_last_quarter, n_5-8, n_9-14 and frequency features for each region). The regions at residues 5–8 and 9–14 represent apparent functional domains described by Abudayyeh et al., Gootenberg et al. and Tambe et al.^4,5,14^. Another set of the features describes distribution of the mismatches across the spacer (min, max, mean, range, IRQ). Spacer/target pairs with a single mismatch were given position values of 0 for calculations of features related to mismatch distribution. To calculate the interquartile range (IRQ) for a set of mismatch positions corresponding to a particular spacer/target pair the quartiles Q1 and Q3 were determined and IRQ was obtained by subtracting Q1 value from Q3 (IQR = Q3 – Q1). The IRQ value reflects the uniformity of distribution of mismatches across the length of the spacer. Values of IRQ close to 14 indicate a uniform distribution of mismatches along the spacer while values much lower than 14 corresponding to mismatches arranged as a single cluster and values much higher than 14 corresponding to mismatches arranged in two separate clusters. Final set of features was related to the protospacer flanking site (PFS) identity (PFS_1, PFS_2) and these features reflected the bases present at the FPS locations of the target sequences.

The results of the Cas13a activity assays using a particular crRNA spacer/target combination were designated as either positive or negative based on the following criteria: a sample was evaluated as negative if the cumulative fluorescent, background subtracted, signal was less or equal of 20% of the maximum signal obtained in the experiment (low performing crRNAs), and positive if it was greater than 20% of the maximum signal obtained in the experiment (medium and high performing crRNAs). According to these criteria, 108 spacer/target pairs were labeled negative and 84 were labeled positive.

### Models and feature importance

Models were built to classify the spacer/target combinations as producing positive or negative assay outcomes. Rule-based models such as RuleFit use ensembles of linear models to construct either classification or regression predictions that have been shown to be comparable in accuracy as the best alternatives^26^. However, their main advantage is in their interpretability, as each rule in the ensemble is a simple statement related to the individual features in the input dataset. This property of RuleFit allows for clear ranking of the relative importance of each feature, and allows to better understand their data and the predictions.

The classification model was generated in R using the Tidymodels series of packages^27^. Rule based Learning Ensembles (RuleFit) were assembled with the XRF package^26^. The number of trees contained in the ensemble was set to 2, maximum depth of the tree was set to 3, and the L1 regularization parameter was set to 0.01; all other parameters were set to defaults.

## Supplementary Information

Supplementary Information 1.

Supplementary Information 2.

Supplementary Information 3.

## Data Availability

All data and code used in this study and not included in the supplementary materials are available through the GitHub repository at https://github.com/NRL-CRISPR/CRISPR-rules.
