# Supplementary material for: Machine learning for design of degenerate Cas13a crRNAs using lassa virus as a model of highly variable RNA target
Source: Sci Rep. 2023 Apr 20;13:6506. doi: 10.1038/s41598-023-33494-4 (PMC10119381; doi:10.1038/s41598-023-33494-4)
Supplement: Supplementary file 1 — Supplementary Information 1. [file 41598_2023_33494_MOESM1_ESM.docx]

Table of Contents

[Table S1. Sequences used as LASV and near neighbor targets 3](#_Toc131443676)

[Figure S1. Taxonomic relationship of LASV and near-neighbor Old World Arenaviruses used to design target sequences used in this study 4](#_Toc131443677)

[LASV virus lineages 5](#_Toc131443678)

[Table S2. Design of the degenerate consensus and target regions. 6](#_Toc131443679)

[Lineage II target sequences 7](#_Toc131443680)

[Lineage IV target sequences 8](#_Toc131443681)

[Figure S2. Echo based workflow 9](#_Toc131443682)

[Figure S3. crRNA performance against their respective target sequences 10](#_Toc131443683)

[Figure S4. Cross-reactivity with non-specific targets. 11](#_Toc131443684)

[Figure S5. Limits of detection (LOD) and impact of background RNA on LOD. 12](#_Toc131443685)

[Figure S6. crRNA #5 performance versus LASV lineages and near neighbors 13](#_Toc131443686)

[Figure S7. crRNA #9 performance versus LASV lineages and near neighbors 14](#_Toc131443687)

[Figure S8. crRNA #29 performance versus LASV lineages and near neighbors 15](#_Toc131443688)

[Figure S9. crRNA #33 performance versus LASV lineages and near neighbors 16](#_Toc131443689)

[Table S3. Description spacer/target dataset features used in RuleFit model. 18](#_Toc131443690)

[Table S4. Top 10 rules from the RuleFit classifier (Watson-Crick paring) 19](#_Toc131443691)

[Figure S10. ROC/AUC graph (Watson-Crick paring) 19](#_Toc131443692)

[Figure S11. Fluorescent signal vs. spacer/target mismatch number 20](#_Toc131443693)

[Table S5. Percentage of assay outcomes classified as Positive in spacer/target pairings with specific mismatch numbers. 21](#_Toc131443694)

[RuleFit classifier model results for the dataset using asymmetric G-U wobble pairing rules. 22](#_Toc131443695)

[Figure S12. Model performance and the most important features 22](#_Toc131443696)

[Figure S13. ROC/AUC graph 22](#_Toc131443697)

[Table S6. Top 10 rules from the RuleFit classifier 23](#_Toc131443698)

[RuleFit classifier model results for the dataset using symmetric G-U wobble pairing rules. 24](#_Toc131443699)

[Figure S14. Model performance and the most important features 24](#_Toc131443700)

[Figure S15. ROC/AUC graph 24](#_Toc131443701)

[Table S7. Top 10 rules from the RuleFit classifier 25](#_Toc131443702)

[Generalized design rules for degenerate crRNAs 26](#_Toc131443703)

[Figure S16. Generalized approach to predictive application of design rules. 26](#_Toc131443704)

[References 27](#_Toc131443705)

**Section 1: crRNA design and screening**

# Table S1. Sequences used as LASV and near neighbor targets

| **Species** | **Isolate** | **LASV lineage** | **Accession #** |
| --- | --- | --- | --- |
| Lassa | Pinneo-NIG-1969 | I | KM822127 |
|  | Nig08-A47 | II | GU481079 |
|  | Nig08-A37 | II | GU481075 |
|  | LASV0217-DELTA-2018 | II | MH887993 |
|  | CSF | III | AH012598 |
|  | ONM-314 | III | KT992433 |
|  | Josiah-GPA | IV | MW004546 |
|  | Guinea Faranah | IV | KU978808 |
|  | Macenta | IV | AY628200 |
|  | AV | V | FR832710 |
|  | Soromba-R | V | KF478762 |
|  | KAK-428 | VI | KT992435 |
|  | Togo/2016/7082 | VII | KU961972 |
| Mopeia | AN20410 | N/A | AY772169 |
| Mobala | Acar 3080 | N/A | DQ328876 |
| Lujo | IGR140 | N/A | JX017362 |
| Dandenong | 0710-2678 | N/A | EU136039 |
| Morogoro | 3017/2004 | N/A | EU914104 |
| Gairo | TZ-27421_L | N/A | NC_026247 |
| Wenzhou | Rn-YCB1 | N/A | KY662262 |
| Lijiang | KS4 | N/A | MF414201 |
| Loie River | R5074 | N/A | KC669693 |
| LCMV | 810935 | N/A | FJ607020 |
| Ippy | Dak An B 188 d | N/A | DQ328878 |

# Figure S1. Taxonomic relationship of LASV and near-neighbor Old World Arenaviruses used to design target sequences used in this study


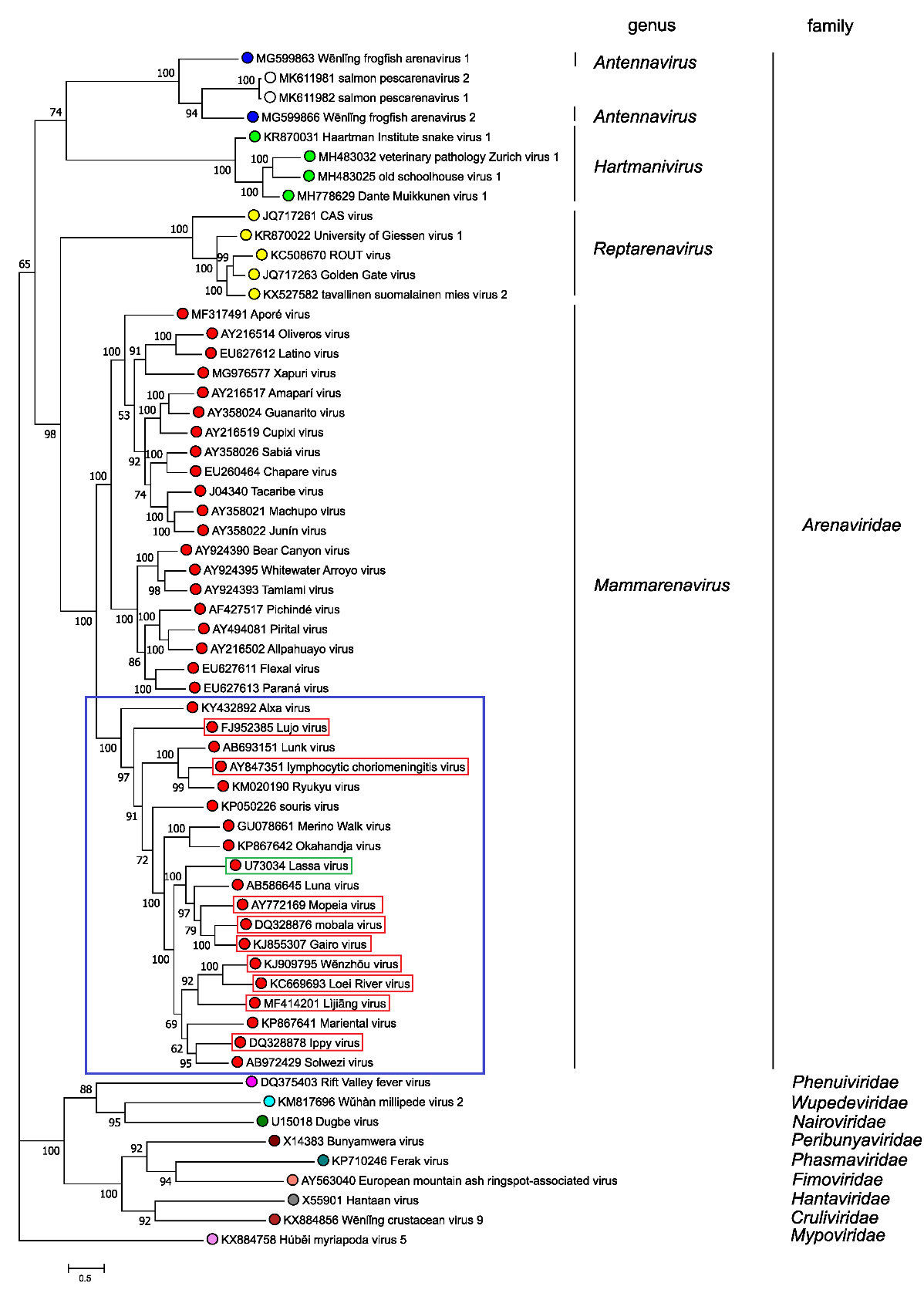


The *Arenaviridae* family taxonomic tree was reproduced from the report of International Committee on Taxonomy of Viruses (ICTV)^1^. The branch of the tree enclosed in blue box includes the Old World Arenavirus (OWA) group. LASV is enclosed in green box and the near-neighbor species used to design the target sequences are enclosed in red boxes. Dandenong virus (closely related to LCMV) and Morogoro (closely related to Mopeia virus) are not shown here.

# LASV virus lineages

LASV, the etiologic agent of Lassa Fever (LF) is known for high degree of variability of its genome with multiple lineages circulating in West Africa. The first comprehensive analysis of the LASV genomic data revealed four main LASV lineages with lineage s I, II and III circulating in Nigeria and lineage IV present in Mano River Union (MRU) region encompassing Guinea, Sierra Leone and Liberia with variation at nucleotide level close to 30% ^1^. More recently, the LASV isolates from Mali and Côte d’Ivoire were proposed to form a separate lineage V ^2^ and isolates from Nigeria and Togo to represent further two lineages, VI and VII respectively ^3,4^. There is a preliminary evidence of additional LASV lineages circulating in Nigeria ^5,6^. Another factor complicating specific detection of LASV, in addition to its diversity, is the presence of several near neighbor *Mammarenavirus* species, most of them not pathogenic for humans ^7^.

# Table S2. Design of the degenerate consensus and target regions.

| **Gene** | **Lineage** | **Sequences aligned** | | **Consensus sequence^1^** | | **crRNA spacers^2^** | **Templates for RNA targets^3^** | |
| --- | --- | --- | --- | --- | --- | --- | --- | --- |
|  |  | **Number** | **Accession numbers** | **Length**  **(nt)** | **Total no. of degenerated bases** | **Number** | **Accession numbers** | **Length**  **(nt)** |
| GPC | II | 4 | GU481068, GU481074, GU481076, GU481078 | 211 | 35 | 37 | GU481074 | 581 |
| L | II | 4 | GU481075, GU481077, GU481079, GU481069 | 370 | 63 | 50 | GU481075 | 714 |
| GPC | IV | 35 | KM406590-KM406623, GU979505 | 280 | 53 | 27 | GU979505 | 353 |
| L | IV | 35 | KM406557-KM406589, AY363906, AY363907, AY363908 | 373 | 103 | 50 | AY363906 | 473 |

^1^ See consensus sequences below.

^2^ Degenerate spacer sequences tiled along the consensus sequences, their numbers vary due to differences of length of target sequences.

^3^ Sequences of DNA templates used as target sequences in crRNA performance assays.

# Lineage II target sequences

**L gene**

Degenerate target region used for spacer design

GTGAGAGTAATTCTGRAGCACTTAGCAARGCYTTRTCYCTAACYAAYTGYACYACAGCAATGTTAAARAAYTTGTGTTTYTAYAGYCAGGARTCRCCTCARTCTTAYAATTCAACTGGYCCTGAYACYGGTAGAYTRAAATTYTCTTTRTCYTACAAGGAACARGTRGGRGGYAAYAGAGAGTTGTACATYGGRGAYYTRAGRACRAAGATGTTYACRAGGCTCATYGARGATTACTTTGAAGCRCTYACATCACAATTRRCRGGCAGYTGYYTAAACAATGAGAARGARTTYGACAATGCCATYTTRTCAATGAARYTRAATGTCTCATCRGCACAYGTATCATAYAGYATGGATCACAGYAAGTGGGG

Synthetic target template (Reverse complement to Genbank accession#: GU979505: 3361-4074; yellow highlight: crRNA target region; green highlight: primer regions)

CAGTGGTGACTAGAACCTTTTTGGACCAAGAATATTTCCAGTGCTTTAAGTCAATTTTATTAGTGATGAATGGGAATAAGCTGATGGGAAGATACTCTCATTATAAGAGTAAGTGTCTGAACTTTAAATTTGATACAGGAAGACTGGCTGATGATGTCAGGATAAGTGAACGTGAGAGTAATTCTGAAGCACTAAGCAAGGCCTTGTCCCTAACCAACTGCACTACAGCAATGTTAAAAAACCTGTGTTTTTACAGTCAAGAATCACCTCAATCTTATAATTCAACTGGCCCTGACACTGGTAGACTAAAATTCTCTTTGTCGTACAAGGAACAAGTGGGAGGCAATAGAGAGTTGTACATCGGGGACTTAAGGACAAAGATGTTTACAAGGCTCATTGAGGATTACTTTGAAGCGCTCACATCACAATTAGCAGGCAGCTGTCTAAACAATGAGAAAGAGTTTGACAATGCCATTTTGTCAATGAAGCTGAATGTCTCATCGGCACATGTATCATATAGCATGGATCACAGCAAGTGGGGCCCTATGATGTGTCCTTTCTTATTTCTAACAATAATTCAAAACCTAATCTTACTTTCTGATGATTTACAAGCTGATTTGAAAGGTAAAGATTACCTATCAACATTATTAACATGGCATATGCACAAAATGGTTGAGATTCCATTTAATGTTGTATCAGCAATGATGAAGTCAT

**GPC gene**

Degenerate target region used for spacer design

AACCTYTCTGATGCACAYAARARGAATCTYTATGAYCATGCYYTRATGAGYATCATYTCAACYTTYCAYTTATCYATTCCYAAYTTTAATCAGTATGAAGCAATGAGYTGTGACTTYAATGGRGGGAARATAAGTGTTCARTACAAYCTYAGYCAYRCYTATGCTGTRGATGCAGCYAACCACTGTGGGACYATYGCCAAYGGYGTYCTTC

Synthetic target template (Genbank accession#: GU979504: 219-799; yellow highlight: crRNA target region; green highlight: primer regions)

ATGTTCAACAACTTACAAGGGTGTTTATGAGCTACAAACTCTGGAGCTAGACATGGCAAGCCTTAATATGACAATGCCCTTATCTTGCACGAAGAACAACAGCCACCATTACATTATGGTCGGAAATGAGACTGGCTTGGAGCTGACTTTAACAAATACAAGTATCATCAACCACAAATTTTGTAACCTCTCTGATGCACATAAAAAGAATCTTTATGATCATGCTTTAATGAGTATCATTTCAACCTTCCACTTATCCATTCCTAACTTTAATCAGTATGAAGCAATGAGTTGTGACTTCAATGGGGGGAAGATAAGTGTTCAGTACAACCTTAGCCACACTTATGCTGTAGATGCAGCCAACCACTGTGGGACTATCGCCAATGGCGTTCTTCAGACTTTTATGAGGATGGCTTGGGGTGGCAGTTATATAGCACTTGATTCCGGAAAGGGGAGTTGGGACTGTATAATGACTTCCTACCAATATTTGATAATCCAAAACACCACTTGGGAAGATCACTGCCAGTTTTCTCGCCCATCCCCTATCGGTTACCTAGGGCTACTGTCACAAAGGACCAGGG

# Lineage IV target sequences

**L gene**

Degenerate target region used for spacer design

AGGGARAGYAAYTCRGARGCACTYAGTAARGCYYTRTCAYTRACAAAYTGYACYACYGCAATGYTRAARAAYYTRTGYTTYTAYAGYCARGARTCRCCACARTCYTAYRAYTCYGTRGGRCCTGAYACRGGRAGRCTYAARTTYTCYTTRTCATAYAARGARCARGTRGGYGGYAAYAGRGARYTRTAYATYGGTGAYCTYAGRACRAARATGTTYACYAGRCTYATAGARGATTAYTTYGAAGCYYTAAGYYTRCAGYTRTCRGGRAGYTGYYTRAAYAAYGARAARGAATTTGARAAYGCYATCCTTTCCATGAARYTRAATGTTTCRYTRGCACAYGTCTCYTATAGTATGGAYCAYAGTAARTGGGGCC

Synthetic target template (Genbank accession#: AY363906; yellow highlight: crRNA target region; green highlight: primer regions)

ATTTCAAGTTTGATATGGGTAAACTCTCTGATGATGTGAGAATCAGTGAGAGGGAGAGCAATTCAGAAGCACTTAGTAAGGCTCTGTCACTGACAAACTGTACCACTGCAATGTTAAAAAACTTGTGTTTTTACAGCCAAGAGTCGCCACAATCTTATGACTCTGTGGGGCCTGATACAGGAAGGCTTAAATTTTCTTTATCATATAAAGAACAAGTAGGTGGTAACAGAGAGTTATATATCGGTGATCTTAGGACAAAGATGTTTACTAGACTTATAGAAGATTACTTTGAAGCCCTAAGCCTACAGTTATCAGGGAGTTGCCTGAACAATGAGAAGGAATTTGAAAATGCTATCCTTTCCATGAAATTGAATGTTTCATTGGCACATGTCTCCTATAGTATGGATCATAGTAAGTGGGGCCCAATGATGTGTCCATTTTTATTCTTGACTGTCTTACAGAATTTAATTTTC

**GPC gene**

Degenerate target region used for spacer design

TTTTGGTTGCGCAATCYAAGYRTCCYAYTYAAAATGGGACARATAGTGACATTYTTCCAGGARGTGCCYCATGTAATAGARGAGGTGATGAACATTGTTCTCATRGCAYTRTCYATTYTRGCAGTGYTGAARGGTYTGTACAAYYTTGCAACATGYGGCCTYRTTGGYTTRRTYAGYTTYCTYYTRYTGTGTGGYAGRTCYTGYTCAACYARYCTTTAYAARGGRGTYTATGAGCTTCAGACTYTRGARYTRAACATGGARACACTCAAYATGACCATGC

Synthetic target template (Genbank accession#: GU979505/6 SL21/SL15; yellow highlight: crRNA target region; green highlight: primer regions)

GCGCACCGGGGATCCTAGGCATTTTTGGTTGCGCAATCCAAGCATCCCATTCAAAATGGGACAAATAGTGACATTCTTCCAGGAAGTGCCTCATGTAATAGAAGAGGTGATGAACATTGTTCTCATTGCACTGTCTATTCTAGCAGTGCTGAAGGGTCTGTACAACTTTGCAACATGTGGCCTCGTTGGTTTGGTCAGCTTCCTCTTGTTGTGTGGCAGGTCTTGCTCAACCAGTCTTTACAAAGGGGTTTATGAGCTTCAGACTTTGGAGCTAAACATGGAGACACTCAACATGACCATGCCTCTCTCTTGCACAAAGAACAACAGTCATCATTACATAATGGTGGGCAATG

# Figure S2. Echo based workflow


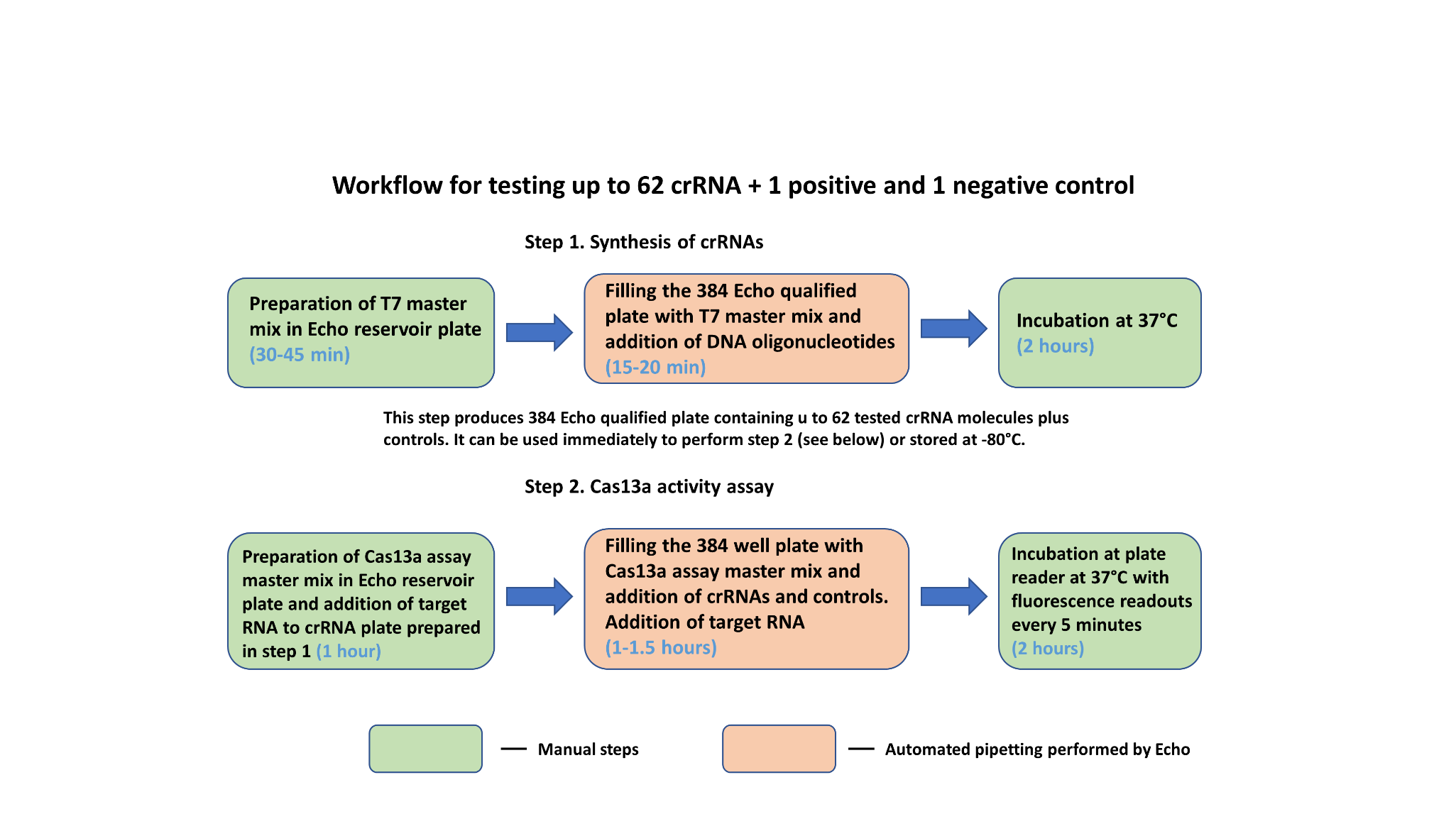


The semi-automated workflow used for testing crRNA performance used in this study using Echo 525 acoustic liquid handler (Beckman Coulter, Indianapolis, IN) and the Plate Reformat software.

# Figure S3. crRNA performance against their respective target sequences

**
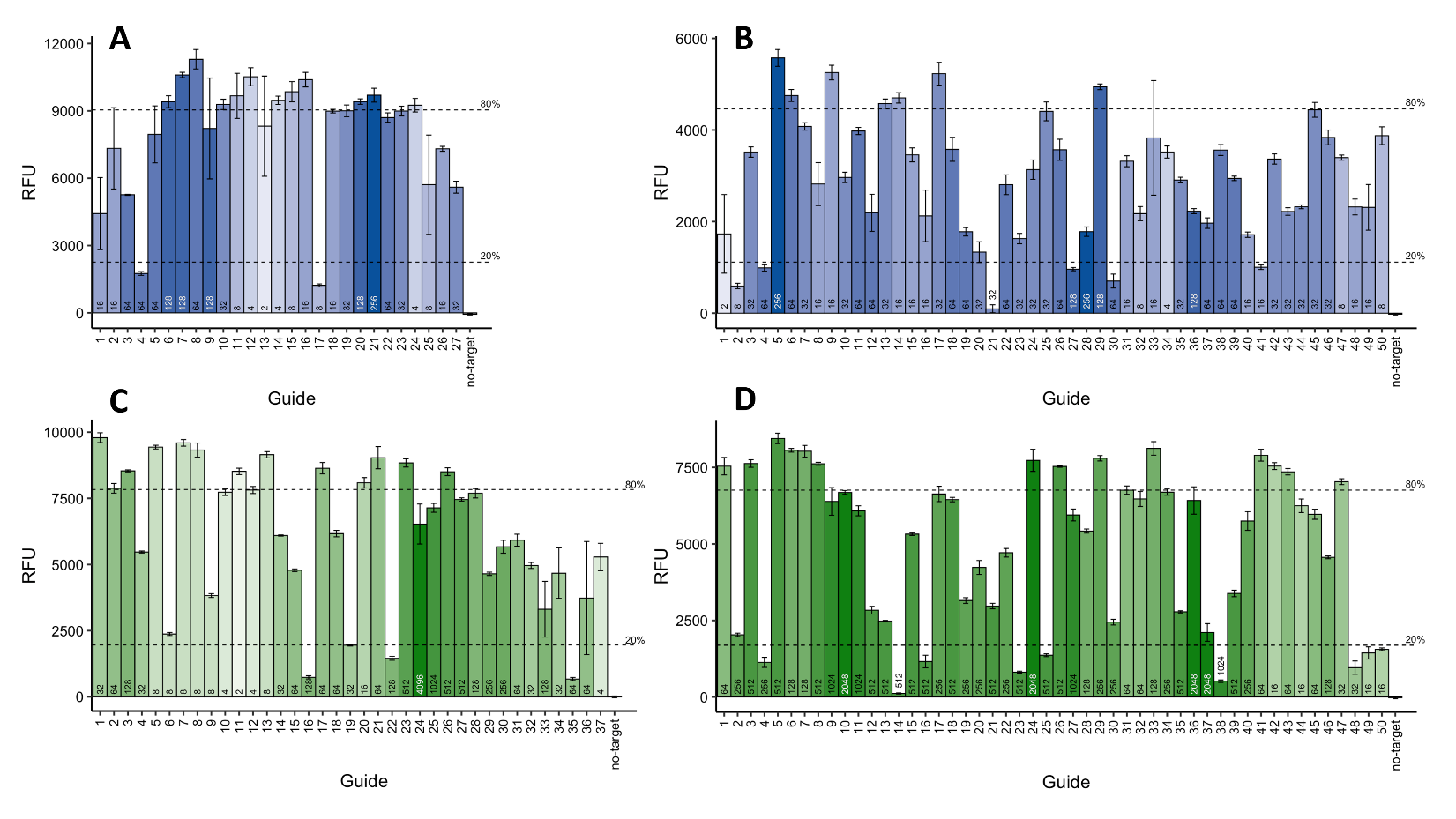
**

Performance testing results for all crRNAs designed for GPC and L targets. The height of the bars reflect the cumulative background subtracted fluorescence signal (RFU) obtained for each of the tested crRNAs with their corresponding targets. The upper (A and B) graphs with blue bars show the results for GPC (A) and L (B) targets designed based on LASV lineage II sequences. The lower (C and D) graphs with green bars show the results for GPC (C) and L (D) targets designed based on LASV lineage IV sequences. The numbers inside the bars and the intensity of their colors reflect degeneracy of the spacer for each of the tested crRNA molecules. The vertical lines denote the signal thresholds used to classify the crRNA performance into low (signal less or equal 20% of the maximum signal), medium (signal higher than 20% but less than 80%) and high (signal equal or higher than 80%).

# Figure S4. Cross-reactivity with non-specific targets.


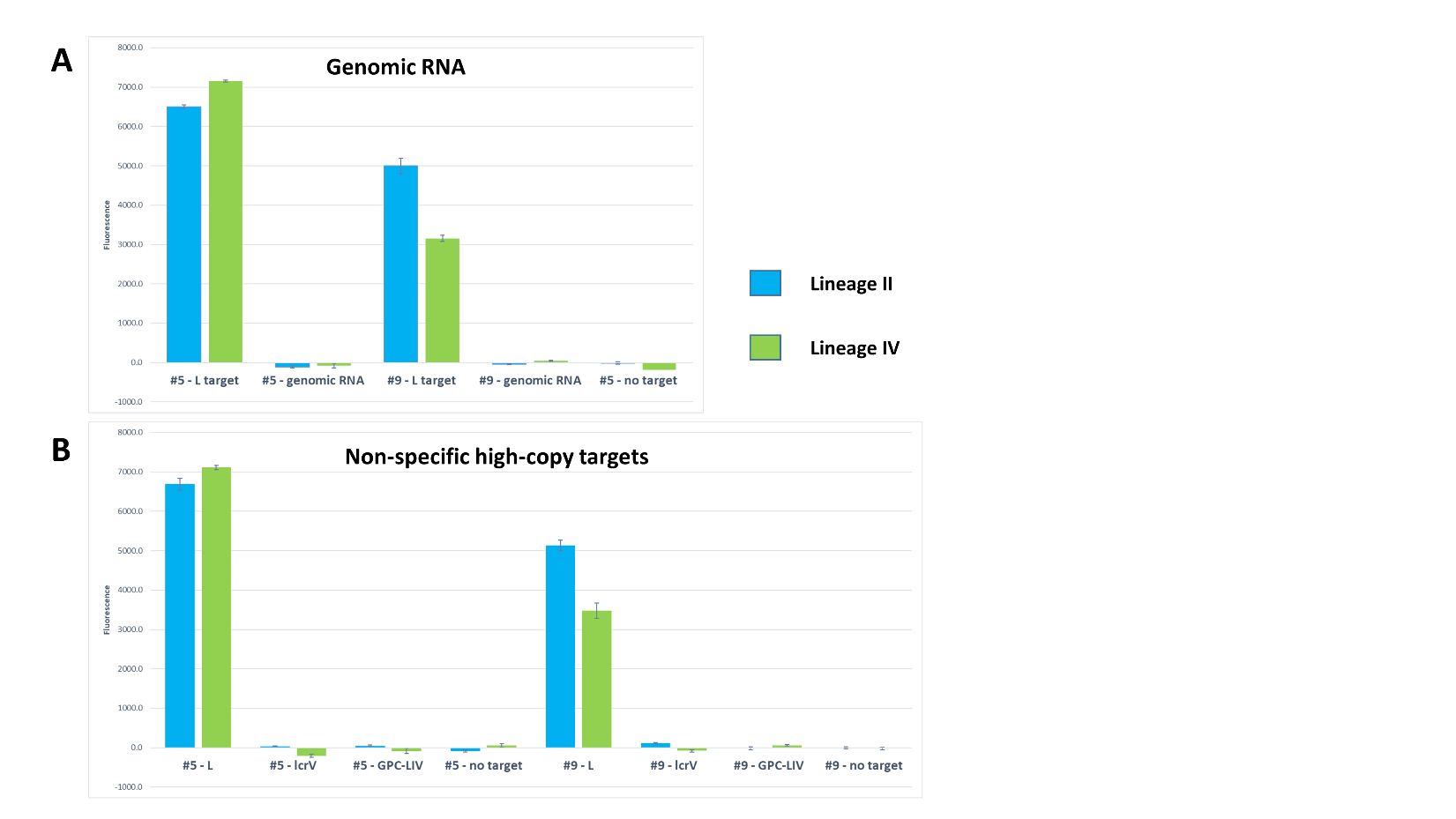


Results of cross-reactivity testing with non-specific targets of selected degenerate crRNAs for L target (#5_LII, #5_LIV, #9_LII and #9_LIV). Panel A – results of cross-reactivity testing with high molecular weight genomic RNA at25ng/µL. Panel B – results of cross-reactivity testing with high copy number, short, non-specific targets *lcrV* and GPC_LIV (at3nM).

# Figure S5. Limits of detection (LOD) and impact of background RNA on LOD.


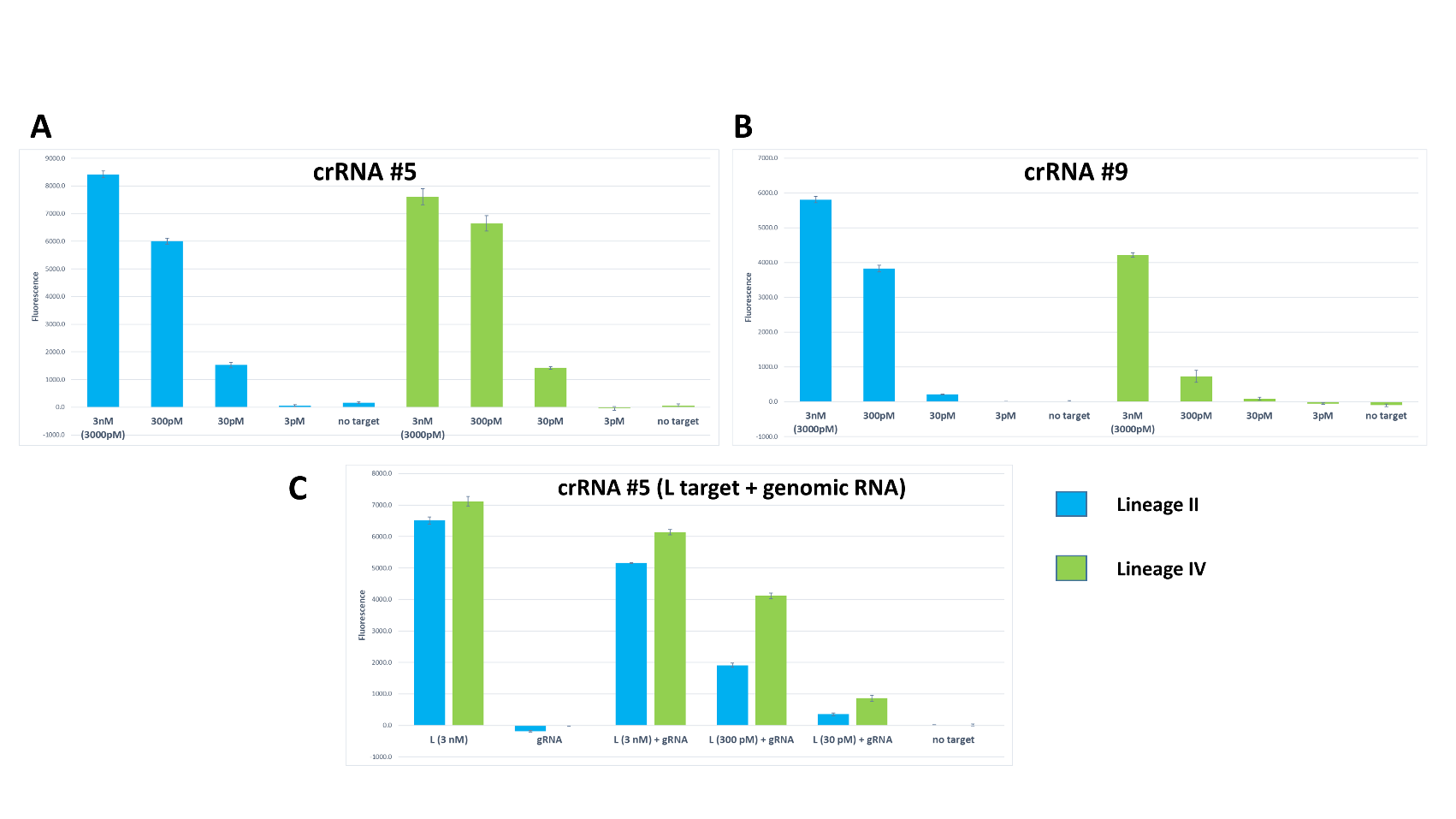


Panel A and B, testing of limit of detection (LOD) for selected crRNAs for L target (#5_LII, #5_LIV, #9_LII and #9_LIV) using 10 fold dilutions of the L target with concentrations ranging from 3nM to 3pM. Panel C, LOD determination for crRNA #5_LII and #5_LIV using 10 fold dilutions of L target with concentrations ranging from 3nM to 3pM in the presence of high molecular weight genomic RNA at25ng/µL.

# Figure S6. crRNA #5 performance versus LASV lineages and near neighbors


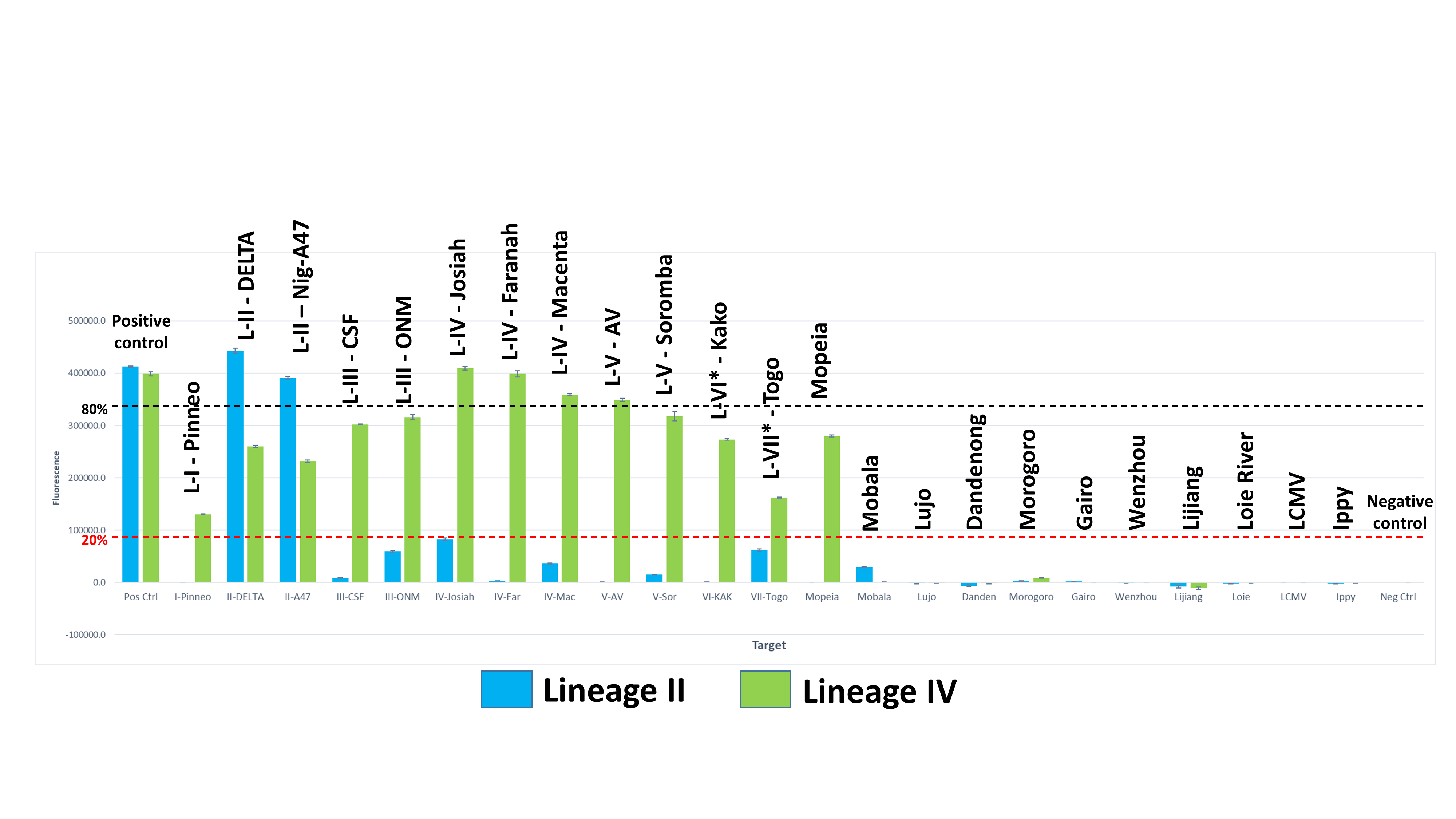


Results of testing of crRNAs #5_LII and #5_LIV with a panel of 12 LASV targets representing all currently known lineages (I-VII) and 11 near-neighbor old world arenavirus (OWA) targets. The height of the bars reflect the cumulative background subtracted fluorescence obtained for each of the tested crRNAs against the specific target. The vertical lines denote the lineage specific signal thresholds used to classify the results of the assays into negative (signal ≤ 20% of the maximum signal), and positive (signal > 20%, which includes medium performing (>20% and <80%) and high performing (≥80%) crRNAs).

# Figure S7. crRNA #9 performance versus LASV lineages and near neighbors


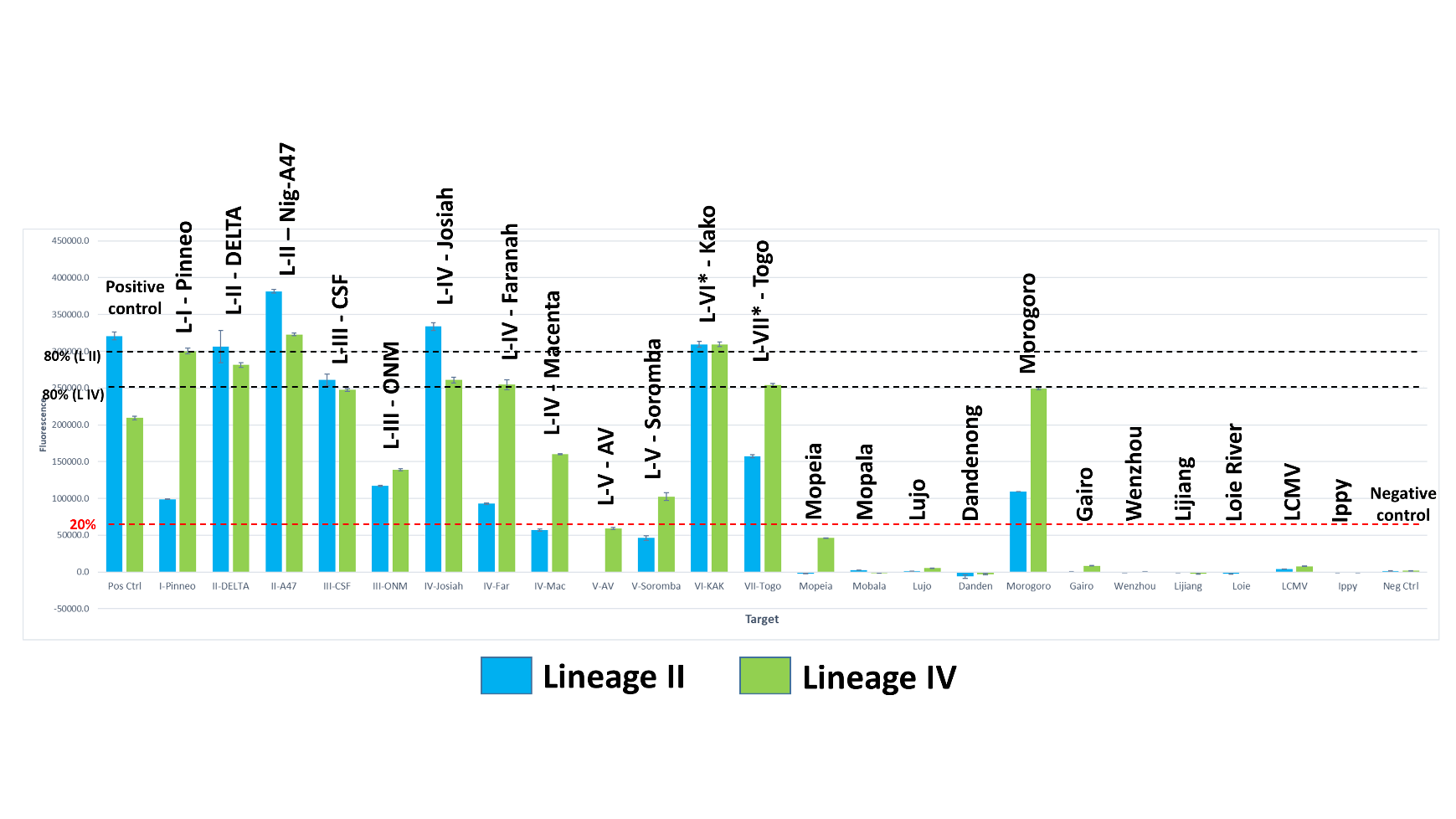


Results of testing of crRNAs #9_LII and #9_LIV with a panel of 12 LASV targets representing all currently known lineages (I-VII) and 11 near-neighbor old world arenavirus (OWA) targets. The height of the bars reflect the cumulative background subtracted fluorescence obtained for each of the tested crRNAs against the specific target. The vertical lines denote the lineage specific signal thresholds used to classify the results of the assays into negative (signal ≤ 20% of the maximum signal), and positive (signal > 20%, which includes medium performing (>20% and <80%) and high performing (≥80%) crRNAs).

# Figure S8. crRNA #29 performance versus LASV lineages and near neighbors


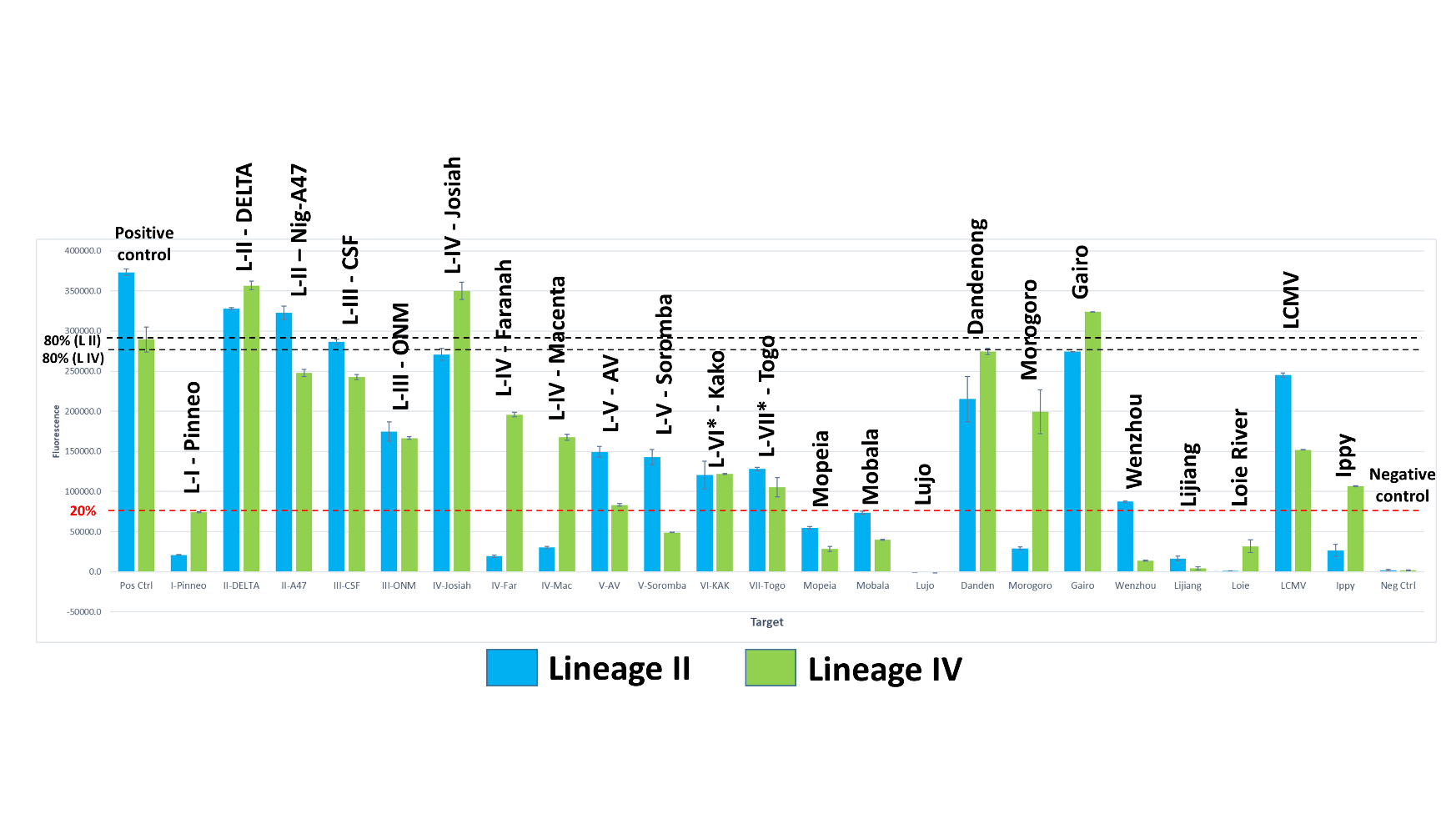


Results of testing of crRNAs #29_LII and #29_LIV with a panel of 12 LASV targets representing all currently known lineages (I-VII) and 11 near-neighbor old world arenavirus (OWA) targets. The height of the bars reflect the cumulative background subtracted fluorescence obtained for each of the tested crRNAs against the specific target. The vertical lines denote the lineage specific signal thresholds used to classify the results of the assays into negative (signal ≤ 20% of the maximum signal), and positive (signal > 20%, which includes medium performing (>20% and <80%) and high performing (≥80%) crRNAs).

# Figure S9. crRNA #33 performance versus LASV lineages and near neighbors


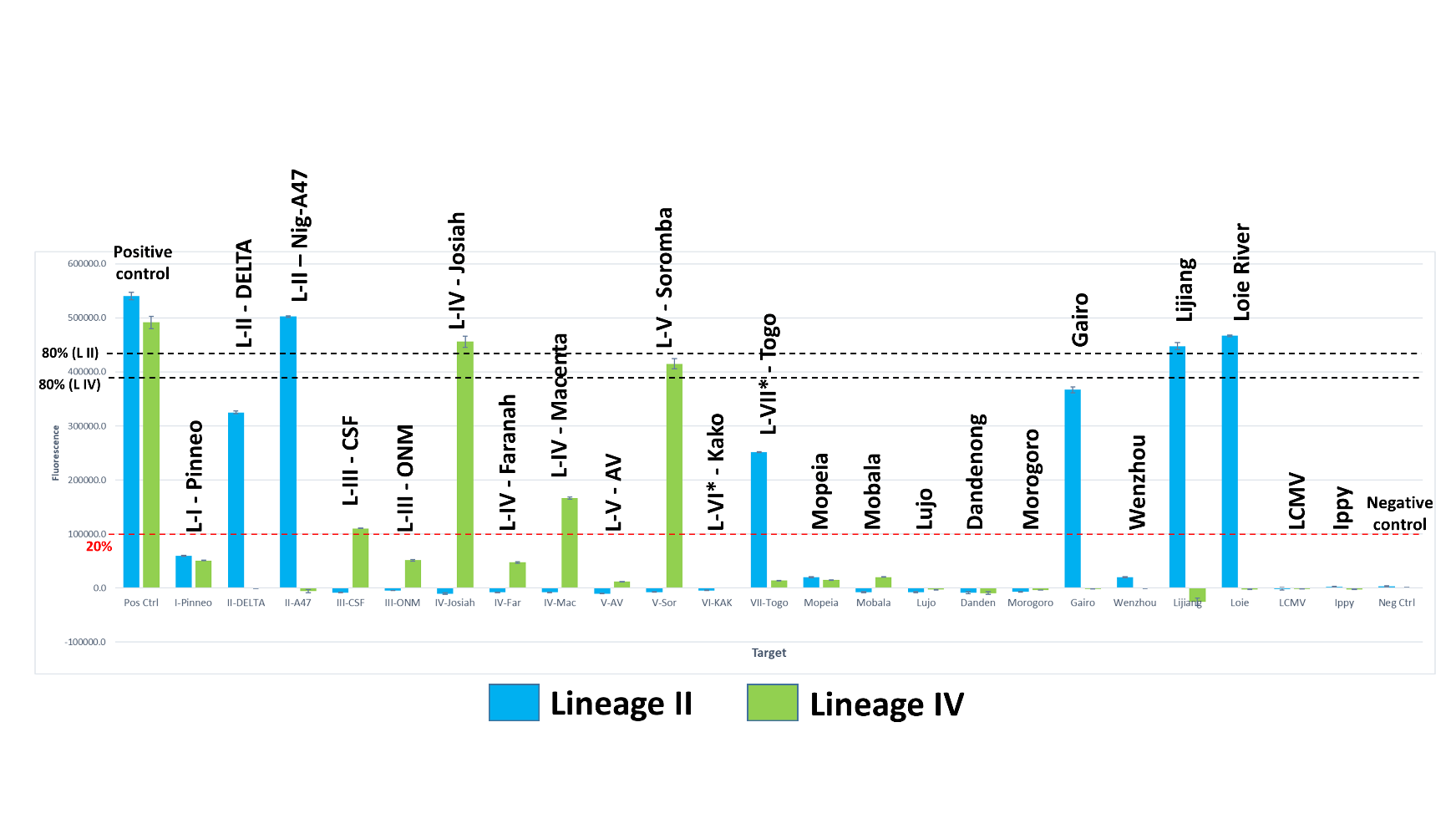


Results of testing of crRNAs #33_LII and #33_LIV with a panel of 12 LASV targets representing all currently known lineages (I-VII) and 11 near-neighbor old world arenavirus (OWA) targets. The height of the bars reflect the cumulative background subtracted fluorescence obtained for each of the tested crRNAs against the specific target. The vertical lines denote the lineage specific signal thresholds used to classify the results of the assays into negative (signal ≤ 20% of the maximum signal), and positive (signal > 20%, which includes medium performing (>20% and <80%) and high performing (≥80%) crRNAs).

**Section 2: RuleFit classifier performance and**

**crRNA design rule development**

# Table S3. Description spacer/target dataset features used in RuleFit model.

| **Feature Name^1^** | **Possible values** | **Observed values^2^** | **Description** |
| --- | --- | --- | --- |
| n | 0-28 | 0-11 | Total number of mismatches between crRNA spacer and the corresponding target sequence |
| n_first_half | 0-14 | 0-5 | Number of mismatches located at spacer positions 1 to 14 |
| n_middle_half | 0-14 | 0-6 | Number of mismatches located at spacer positions 7 to 21 |
| n_last_half | 0-14 | 0-7 | Number of mismatches located at spacer positions 15 to 28 |
| n_first_quarter | 0-7 | 0-5 | Number of mismatches located at spacer positions 1 to 7 |
| n_last_quarter | 0-7 | 0-7 | Number of mismatches located at spacer positions 21 to 28 |
| region_5_8 | 0-4 | 0-3 | Number of mismatches located at spacer positions 5 to 8 |
| region_9_14 | 0-6 | 0-3 | Number of mismatches located at spacer positions 9 to 14 |
| freq_n_first_half | 0-1 | 0-0.36 | n_first_half divided by 14 |
| freq_n_middle_half | 0-1 | 0-0.43 | n_middle_half divided by 14 |
| freq_n_last_half | 0-1 | 0-0.5 | n_last_half divided by 14 |
| freq_n_first_quarter | 0-1 | 0-0.71 | n_first_quarter divided by 7 |
| freq_n_last_quarter | 0-1 | 0-1 | n_last_quarter divided by 7 |
| freq_region_5_8 | 0-1 | 0-0.75 | freq_region_5_8 divided by 4 |
| freq_region_9_14 | 0-1 | 0-0.5 | freq_region_9_14 divided by 6 |
| min | 0-28 | 0-27 | Spacer position of a mismatch that is nearest to the crRNA hairpin (closest to the 5’ end of crRNA). |
| max | 0-28 | 0-28 | Spacer position of a mismatch that is nearest to the 3’ end of crRNA. |
| mean | 0-28 | 0-27 | Mean value calculated for the spacer position of all mismatches. |
| IQR | 0-28 | 0-21 | The mismatch position Q1 and Q3 quartiles are calculated using the all mismatches between the crRNA spacer and corresponding target sequence. IQR = Q3 – Q1. |
| range | 0-28 | 27 | Distance between the furthest apart mismatches  (range = max – min) |
| PFS_1^3^ | 1=A, 2=U, 3=C, 4=G | 1-4 | Protospacer flanking site #1 - nucleotide in the target sequence directly adjacent to the protospacer sequence |
| PFS_2^3^ | 1=A, 2=U, 3=C, 4=G | 1-4 | Protospacer flanking site #2 - nucleotide in the target sequence directly adjacent to the protospacer sequence |

^1^ See main manuscript, Figure 1B for numbering of nucleotide positions in spacer sequence and description of the spacer regions used as features.

^2^ Value ranges seen in the in the analyzed dataset.

^3^ For PFS features the nucleotides were encoded as numeric values for use with the models.

# Table S4. Top 10 rules from the RuleFit classifier (Watson-Crick paring)

| **Rule** | **Effect estimate^1^** |
| --- | --- |
| ( IQR >= 14.375 ) & ( n = 4 ) | 2.47 |
| ( IQR >= 6.25 ) & ( n < 3.5 ) & ( pfs_1 < 3.5 ) | 2.04 |
| ( IQR < 6.25 ) & ( n < 3 ) | 1.72 |
| ( min >= 3.5 ) & ( n = 4 ) | -1.53 |
| ( IQR < 6.25 ) & ( n = 3 ) | -1.27 |
| ( n >= 4 ) | -1.17 |
| ( min < 3.5 ) & ( n = 4 ) | 0.93 |
| ( n ) | -0.70 |
| ( IQR >= 1.25 ) & ( n < 3.5 ) & ( n_first_half < 1.5 ) | 0.68 |
| ( n_last_quarter ) | -0.20 |

^1^A negative effect estimate indicates the rule supports Negative classification.

# Figure S10. ROC/AUC graph (Watson-Crick paring)


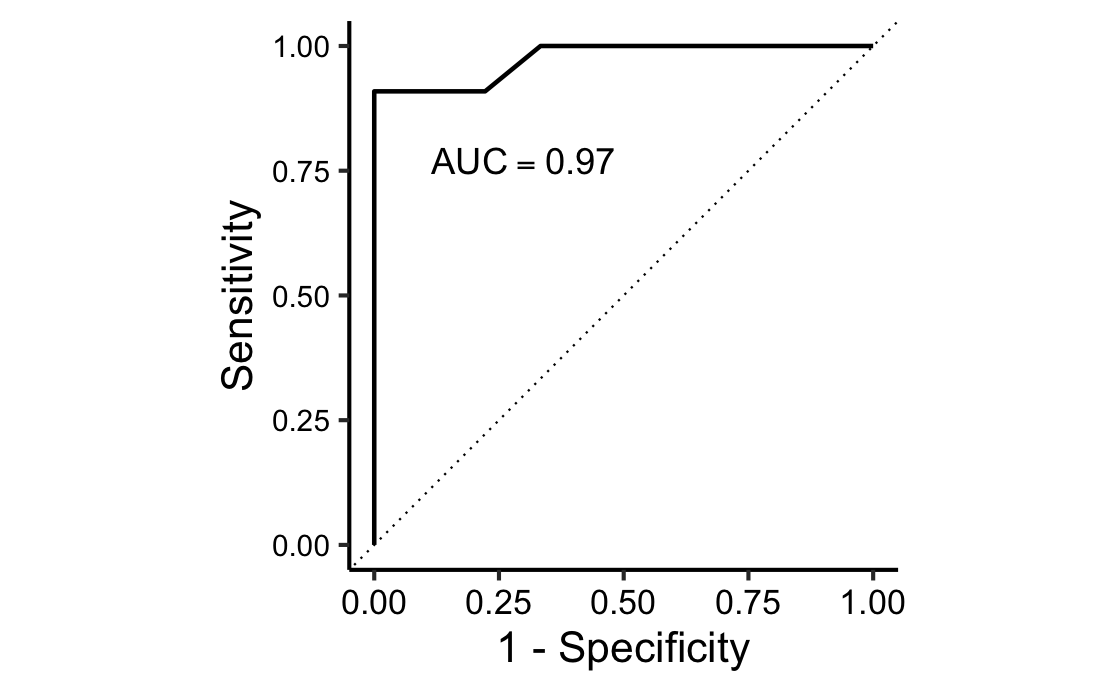


# Figure S11. Fluorescent signal vs. spacer/target mismatch number


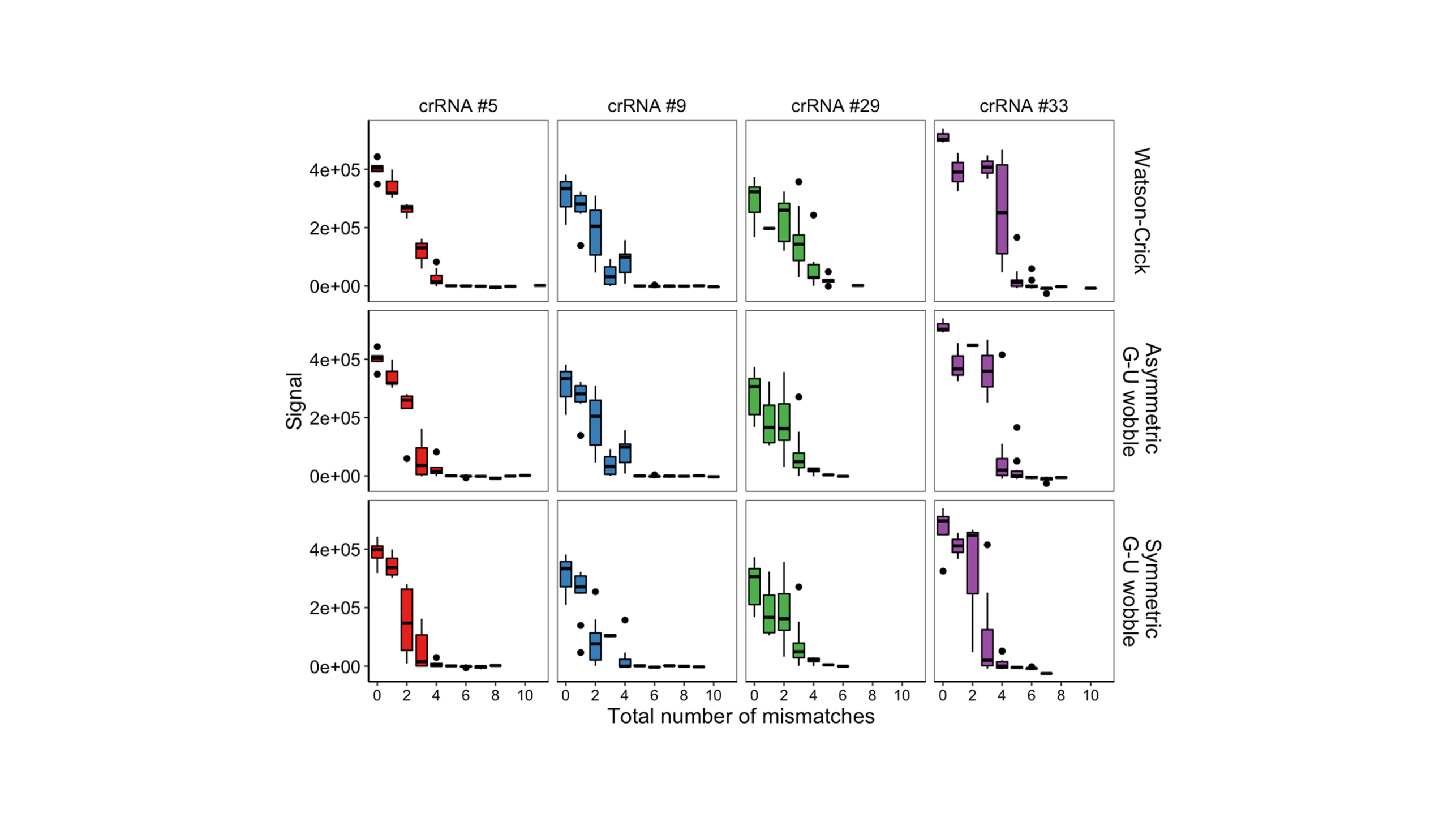


Relationship between the number of mismatches of the spacer/target pairs and the cumulative background subtracted fluorescence signal obtained in and Ca13a assay for all the spacer/target pairs tested for L gene crRNAs #5, #9, #29 and #33.for. Watson-Crick pairing rules (upper panel), asymmetric G-U wobble paring rules (middle panel) and symmetric G-U wobble paring rules (lower panel)

# Table S5. Percentage of assay outcomes classified as Positive in spacer/target pairings with specific mismatch numbers.

| **Number of mismatches** | **Percentage of Positive assay outcomes** | | |
| --- | --- | --- | --- |
|  | **Watson-Crick** | **Asymetric**  **G-U wobble** | **Symetric**  **G-U wobble** |
| 0 | 100 | 100 | 100 |
| 1 | 100 | 100 | 96 |
| 2 | 90 | 87 | 69 |
| 3 | 73 | 33 | 34 |
| 4 | 32 | 20 | 3 |
| 5 | 4 | 4 | 0 |
| 6 | 0 | 0 | 0 |
| 7 | 0 | 0 | 0 |
| 8 | 0 | 0 | 0 |
| 9 | 0 | 0 | 0 |
| 10 | 0 | 0 | n/a^1^ |
| 11 | 0 | n/a^1^ | n/a^1^ |

^1^n/a = no spacer/target pairings with this number of mismatches exist for this dataset.

# RuleFit classifier model results for the dataset using asymmetric G-U wobble pairing rules.

## Figure S12. Model performance and the most important features


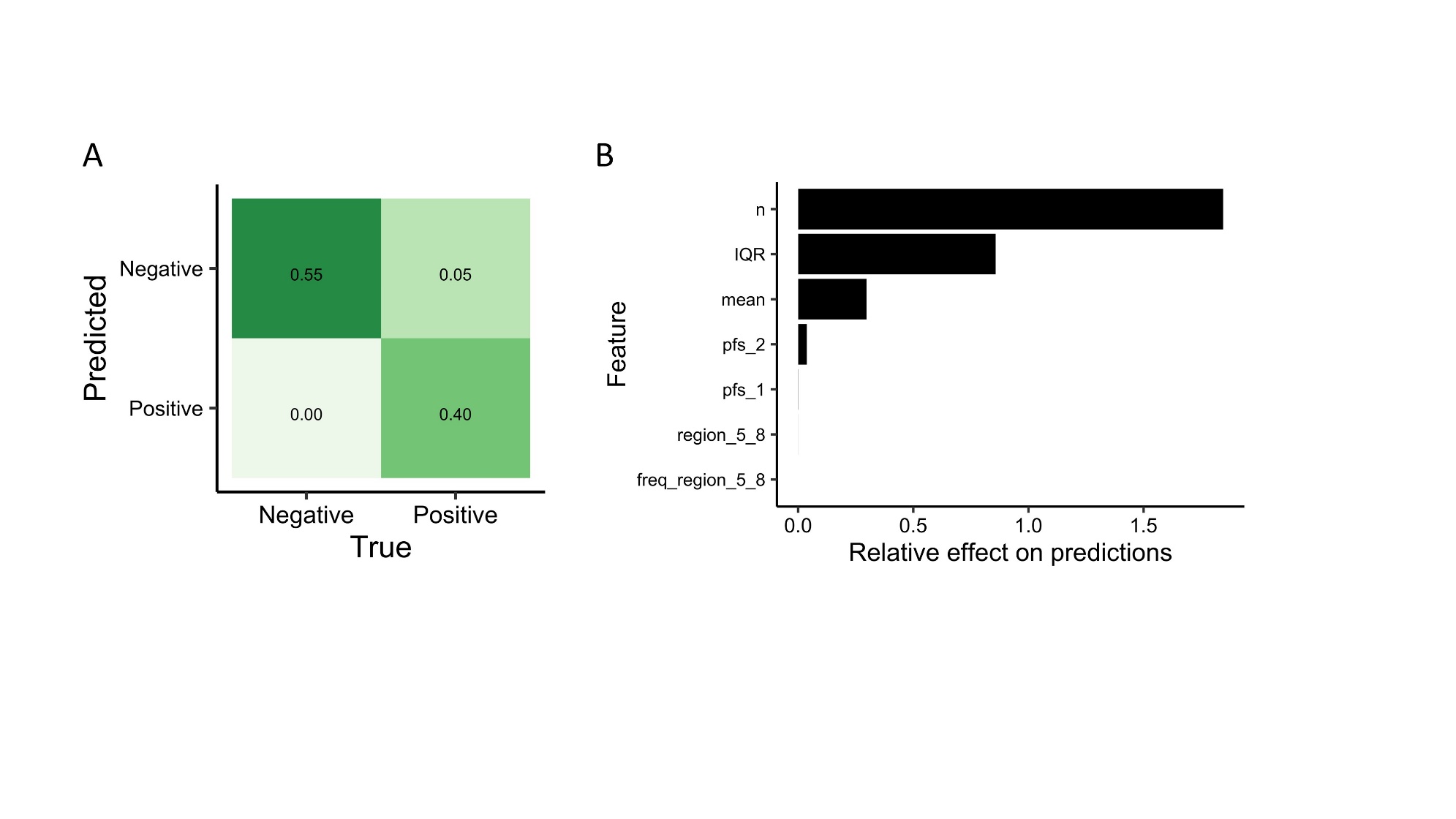


RuleFit classifier model performance for Watson-Crick base pairing. A – confusion matrix showing percentages of actual assay outcomes versus outcomes produced by the classifier model, B – relative effect of features on model predictions – n: number of mismatches, IQR – interquartile range for mismatch positions (characterizes distribution of mismatches with IRQ=14 indicating even distribution and IRQ>14 and IRQ<14 indicating clustering), mean – mean position of a mismatch, PFS_1 and PFS_2 – nucleotide at protospacer flanking site #1 and site #2, remaining features corresponding to various regions of crRNA as described in main manuscript, Figure 1B.

## Figure S13. ROC/AUC graph


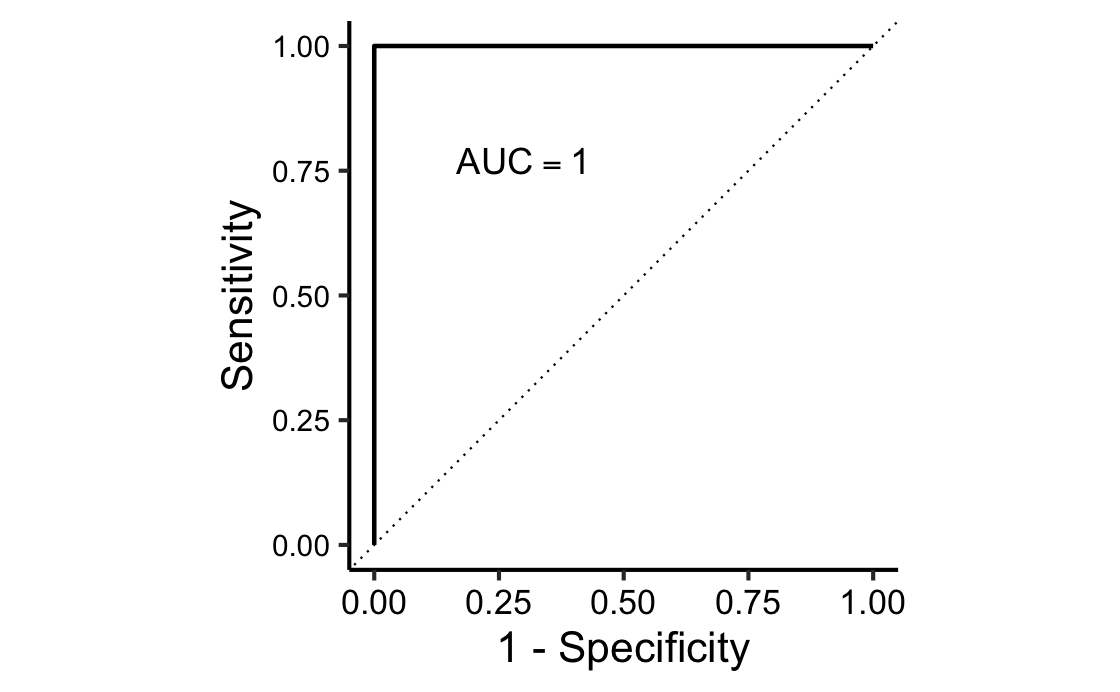


## Table S6. Top 10 rules from the RuleFit classifier

| **Rule** | **Effect estimate^1^** |
| --- | --- |
| ( IQR >= 5 ) & ( mean < 12.75 ) & ( n < 2.5 ) | -1.78 |
| ( n < 2.5 ) | 1.76 |
| ( IQR < 8.375 ) & ( n < 5 ) & ( n >= 3 ) | -1.71 |
| ( IQR >= 8.375 ) & ( n < 5 ) & ( n >= 3 ) | 1.37 |
| ( n ) | -1.08 |
| ( IQR < 5 ) & ( n < 2.5 ) | 0.28 |
| ( pfs_2 ) | 0.23 |
| ( pfs_1 ) | 0.004 |
| ( region_5_8 ) | 0.001 |
| ( freq_region_5_8 ) | 2.15E-15 |

^1^A negative effect estimate indicates the rule supports Negative classification.

# RuleFit classifier model results for the dataset using symmetric G-U wobble pairing rules.

## Figure S14. Model performance and the most important features


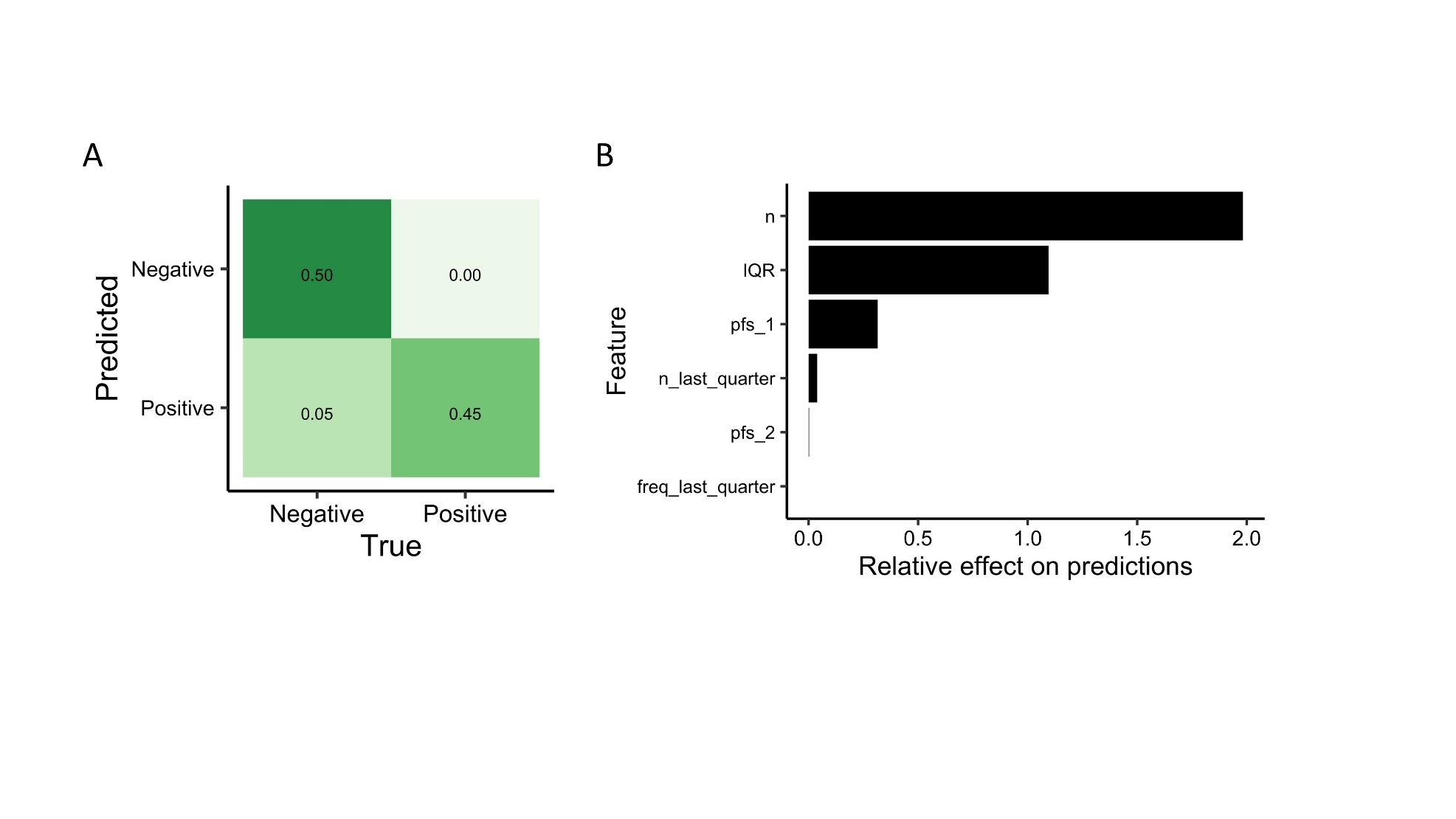


RuleFit classifier model performance for Watson-Crick base pairing. A – confusion matrix showing percentages of actual assay outcomes versus outcomes produced by the classifier model, B – relative effect of features on model predictions – n: number of mismatches, IQR – interquartile range for mismatch positions (characterizes distribution of mismatches with IRQ=14 indicating even distribution and IRQ>14 and IRQ<14 indicating clustering), PFS_1 and PFS_2 – nucleotide at protospacer flanking site #1 and site #2, remaining features corresponding to various regions of crRNA as described in main manuscript, Figure 1B.

## Figure S15. ROC/AUC graph


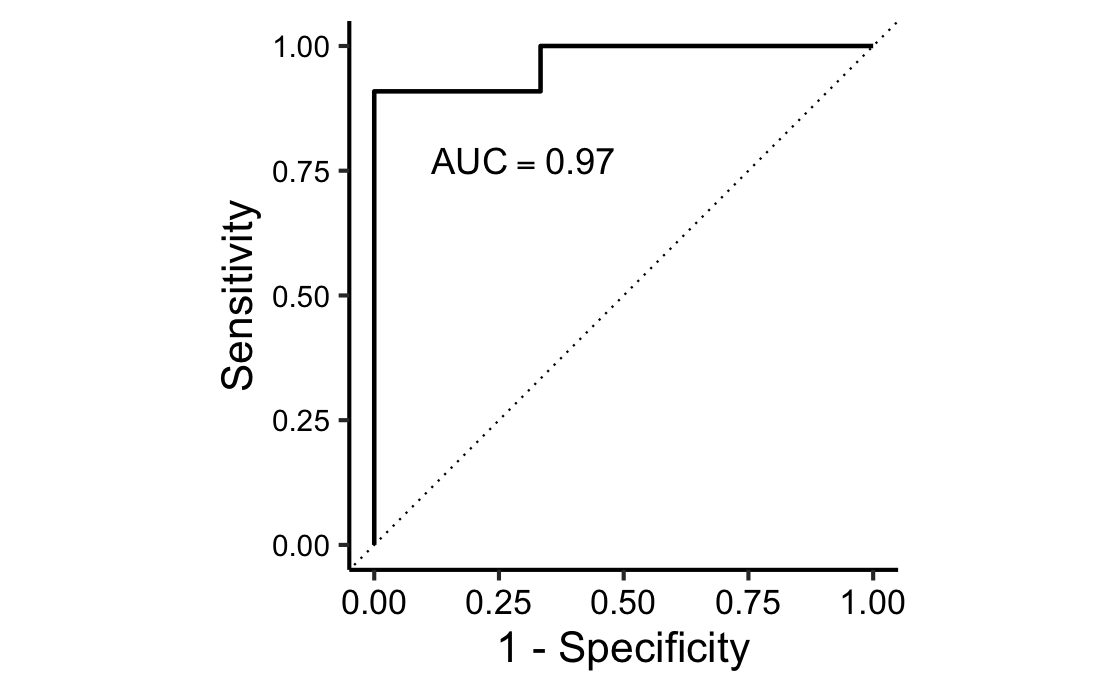


## Table S7. Top 10 rules from the RuleFit classifier

| **Rule** | **Effect estimate^1^** |
| --- | --- |
| ( IQR < 6.5 ) & ( n = 3 ) | -2.54 |
| ( n = 3 ) & ( pfs_1 >= 1.5 ) | 2.21 |
| ( IQR < 5 ) & ( IQR >= 2.75 ) & ( n < 2.5 ) | 1.71 |
| ( n ) | -1.59 |
| ( IQR < 9.25 ) & ( IQR >= 1.25 ) & ( n < 2.5 ) | 0.76 |
| ( n_last_quarter ) | -0.28 |
| ( IQR >= 6.5 ) & ( n = 3 ) | 0.15 |
| ( pfs_2 ) | 0.02 |
| ( IQR >= 9.25 ) & ( n < 2.5 ) | -0.02 |
| ( freq_last_quarter ) | -4.37E-14 |

^1^A negative effect estimate indicates the rule supports Negative classification.

# Generalized design rules for degenerate crRNAs

## Figure S16. Generalized approach to predictive application of design rules.

(1) Assemble and align representative set of sequences for both “inclusion” (taxon of interest) and “exclusion” (near neighbors for which sub-threshold signals are required); (2) Scan genome assemblies of “inclusion” sets to locate candidate nucleotide regions that can be populated with degenerate nucleotides to cover the entire inclusion set while still satisfying degenerate guide RNA design rules. If multiple guides are needed for a given inclusion taxon, identify those. (3) Apply guide design rules to identify any possible false positive signal generation by scanning “exclusion” taxon sequences. Eliminate those candidate guides if detected. (4) Perform limited high-throughput screening of remaining candidate degenerate guides to experimentally confirm signal generation and lack of cross-reactivity.

# References

1 Bowen, M. D. *et al.* Genetic diversity among Lassa virus strains. *J Virol* **74**, 6992-7004 (2000).

2 Manning, J. T., Forrester, N. & Paessler, S. Lassa virus isolates from Mali and the Ivory Coast represent an emerging fifth lineage. *Front Microbiol* **6**, 1037, doi:10.3389/fmicb.2015.01037 (2015).

3 Whitmer, S. L. M. *et al.* New Lineage of Lassa Virus, Togo, 2016. *Emerg Infect Dis* **24**, 599-602, doi:10.3201/eid2403.171905 (2018).

4 Olayemi, A. *et al.* New Hosts of The Lassa Virus. *Sci Rep* **6**, 25280, doi:10.1038/srep25280 (2016).

5 Ehichioya, D. U. *et al.* Phylogeography of Lassa Virus in Nigeria. *J Virol* **93**, doi:10.1128/JVI.00929-19 (2019).

6 Ehichioya, D. U. *et al.* Current molecular epidemiology of Lassa virus in Nigeria. *J Clin Microbiol* **49**, 1157-1161, doi:10.1128/JCM.01891-10 (2011).

7 Gryseels, S. *et al.* Gairo virus, a novel arenavirus of the widespread *Mastomys natalensis*: Genetically divergent, but ecologically similar to Lassa and Morogoro viruses. *Virology* **476**, 249-256, doi:10.1016/j.virol.2014.12.011 (2015).
